# Multi-Level Coupled-Cluster Description of Crystal Lattice Energies

**DOI:** 10.1021/acs.jctc.5c00428

**Published:** 2025-05-29

**Authors:** Krystyna Syty, Grzegorz Czekało, Khanh Ngoc Pham, Marcin Modrzejewski

**Affiliations:** † 49605 University of Warsaw Faculty of Chemistry, Pasteura 1, 02-093 Warsaw, Poland; ‡ Department of Chemical Physics and Optics, Faculty of Mathematics and Physics, 37740 Charles University, Ke Karlovu 3, CZ-12116 Prague 2, Czech Republic

## Abstract

The many-body expansion (MBE) of the lattice energy enables an ab initio description of molecular solids using correlated wave function approximations. However, the practical application of MBE requires computing the large number of *n*-body contributions efficiently. To this end, we employ a multi-level coupled-cluster approach which adapts the approximation level based on interaction type and intermolecular distance. The high-level method, including connected triple excitations, is applied only to monomer relaxation and dimer interactions roughly within the first and second coordination shells. Long-range dimers and trimers are treated using a simplified coupled-cluster description based on the random-phase approximation (RPA). A key feature is an energy correction which mitigates the underbinding error of the base RPA. Convergence to the bulk limit is accelerated by the addition of the periodic Hartree–Fock correction. The proposed approach is validated against recent diffusion Monte Carlo reference data for the X23 data set, achieving a mean absolute error of 3.1 kJ/mol, i.e., chemical accuracy for absolute lattice energies.

## Graphical abstract

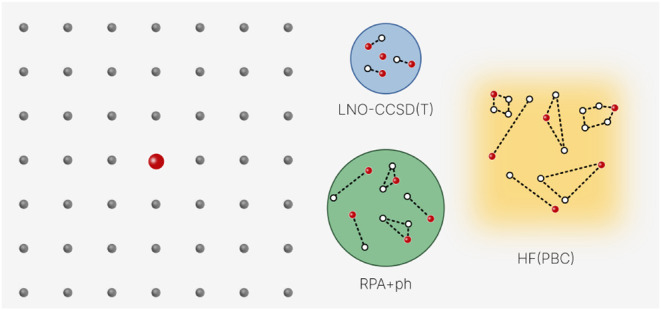

## 1 Introduction

First-principles crystal structure prediction (CSP) of molecular solids from the structures of individual molecules is one of the grand challenges of materials science.^1–3^ A reliable CSP would be a tool to predict the energy landscape of new compounds and to detect kinetically protected metastable states.^4,5^ Those capabilities are relevant for computer-guided design of organic molecular materials and pharmaceuticals.^6^

Correct energetic ordering of noncovalent systems is a key challenge in CSP.^4,5^ This work focuses on the static lattice energy, *E*_latt_, which typically varies by only a few kilojoules per mole between competing polymorphs of organic molecular crystals.^7^ Slight differences in *E*_latt_ arise from, e.g., interplay between intra- and intermolecular interactions^8^ and nonadditive effects.^8^ As a result, qualitative predictions of this physical quantity require sophisticated models of dynamic electron correlation.

Dispersion-corrected density functional theory (DFT), typically implemented with periodic boundary conditions (PBC), has become the standard approach for electronic-structure simulations of molecular solids.^9–15^ A careful selection of the exact exchange admixture and the dispersion model can lead to chemically accurate lattice energies.^16^ However, in the cases where DFT methods disagree with each other or deviate from experiment, such as the conformational polymorphs of *ortho*-acetamidobenzamide and ROY,^8^ wave function methods are preferred due to their systematic convergence to the exact result.^17^

There are two distinct ways of how correlated wave function methods are applied in solids: using PBC^18–20^ or by employing the many-body expansion (MBE).^21,22^ Diffusion Monte Carlo (DMC),^20,23,24^ among PBC-based methods, provides most of the benchmark lattice energies available to date.^20,24^ However, the high computational cost of tightly converged DMC results limits the routine applicability of this approach.^23^

The essence of the MBE approach is to describe periodic systems with the well-established Gaussian basis-set methods designed for molecules.^25,26^ Consider a molecular crystal with a single symmetry-unique reference molecule in the unit cell (denoted as ref). The lattice energy of this system can be expressed as a sum of interaction energies in *n*-body clusters, arranged according to the cluster size

1 $$E_{\mathrm{latt}}=\Delta E_{\mathrm{ref}}+\frac{1}{2}\sum_{i}\Delta^{2}E_{\mathrm{ref},i}+\frac{1}{3}\sum_{i>j}\Delta^{3}E_{\mathrm{ref},i,j}+\cdot \cdot \cdot$$

The first term on the rhs is the monomer relaxation energy

2 $$\Delta E_{\mathrm{ref}}=E_{\mathrm{ref}}\left(\mathrm{crystal}\right)-E_{\mathrm{ref}}\left(\mathrm{isolated molecule}\right)$$

The contributions that follow describe the noncovalent interactions between the reference molecule and its neighbors: the pairwise interaction energy

3 $$\Delta^{2}E_{\mathrm{ref},i}=E_{\mathrm{ref},i}-E_{\mathrm{ref}}-E_{i}$$

the three-body nonadditive interaction energy

4 $$\Delta^{3}E_{\mathrm{ref},i,j}=E_{\mathrm{ref},i,j}-\Delta^{2}E_{\mathrm{ref},i}-\Delta^{2}E_{\mathrm{ref},j}-\Delta^{2}E_{i,j}-E_{\mathrm{ref}}-E_{i}-E_{j}$$

and higher-order nonadditive contributions which make the expansion exactly approach the bulk limit.^22^ The computation of the high *n*-body terms is hard,^27^ but can be done implicitly by including a low-level periodic correction.^28–31^

An advantage of MBE is its compatibility with multi-level wave function approximations. For example, the expensive coupled-cluster method with singles, doubles, and perturbative triples, CCSD­(T),^32,33^ can be limited to short-distance dimers.^28,34–36^

The challenge that remains is an efficient description of the remaining long-distance dimer interactions and the thousands of nonadditive contributions.^37^ The approximations used for these contributions must be substantially cheaper than conventional coupled-cluster methods, but still capture the essential interactions in many-body molecular assemblies.^38^ To this end, second-order Møller–Plesset (MP2) theory supplemented with a dispersion correction has been the method of choice.^21,36,37,39,40^ However, MP2 catastrophically overestimates the interactions between polarizable π-electron systems, where Møller–Plesset perturbation theory diverges.^41–43^

An alternative approach which is more universal than Møller–Plesset theory, but less expensive than established coupled-cluster methods is the random-phase approximation (RPA).^38,44,45^ RPA can be thought of as a coupled-cluster approximation where the doubles amplitude equation retains only the direct ring contributions^46^ and can be solved in a closed form.^47^ The RPA interaction energy correctly reduces to the Casimir-Polder dispersion energy at long separations and accounts for *n*-body dispersion interactions.^38,44,45,48^ Those features make RPA and beyond-RPA methods^47,49^ good candidates to apply in the expensive, many-body part of MBE.

This work presents a multi-level MBE for molecular crystals which combines recent advances in high- and low-level coupled-cluster methods: the local natural orbital^50–52^ (LNO) approximation of CCSD­(T) and RPA with coupled-cluster corrections.^47^ To account for long-range many-body polarization effects, which are unfeasible for large systems within pure MBE, the coupled-cluster description is augmented with periodic Hartree–Fock, HF­(PBC). Validation against canonical CCSD­(T) and DMC data, which is presented in the numerical section, enables a detailed assessment of key methodological components: the accuracy of the LNO approximation, the impact of beyond-RPA corrections, and the role of the HF­(PBC) correction in converging toward accurate lattice energies.

## 2 Theory

### 2.1 Switchover between Levels of Theory

To map a molecular cluster onto an appropriate approximation level, we determine its characteristic distance, *R*, as follows. For a dimer, *R* is the shortest atom–atom separation between two molecules. For a trimer, *R* is the largest of the three intermolecular distances between its constituents. This definition allows us to apply different quantum-chemical approaches in different parts of the system, as shown in Figure 1.

**Figure 1:**
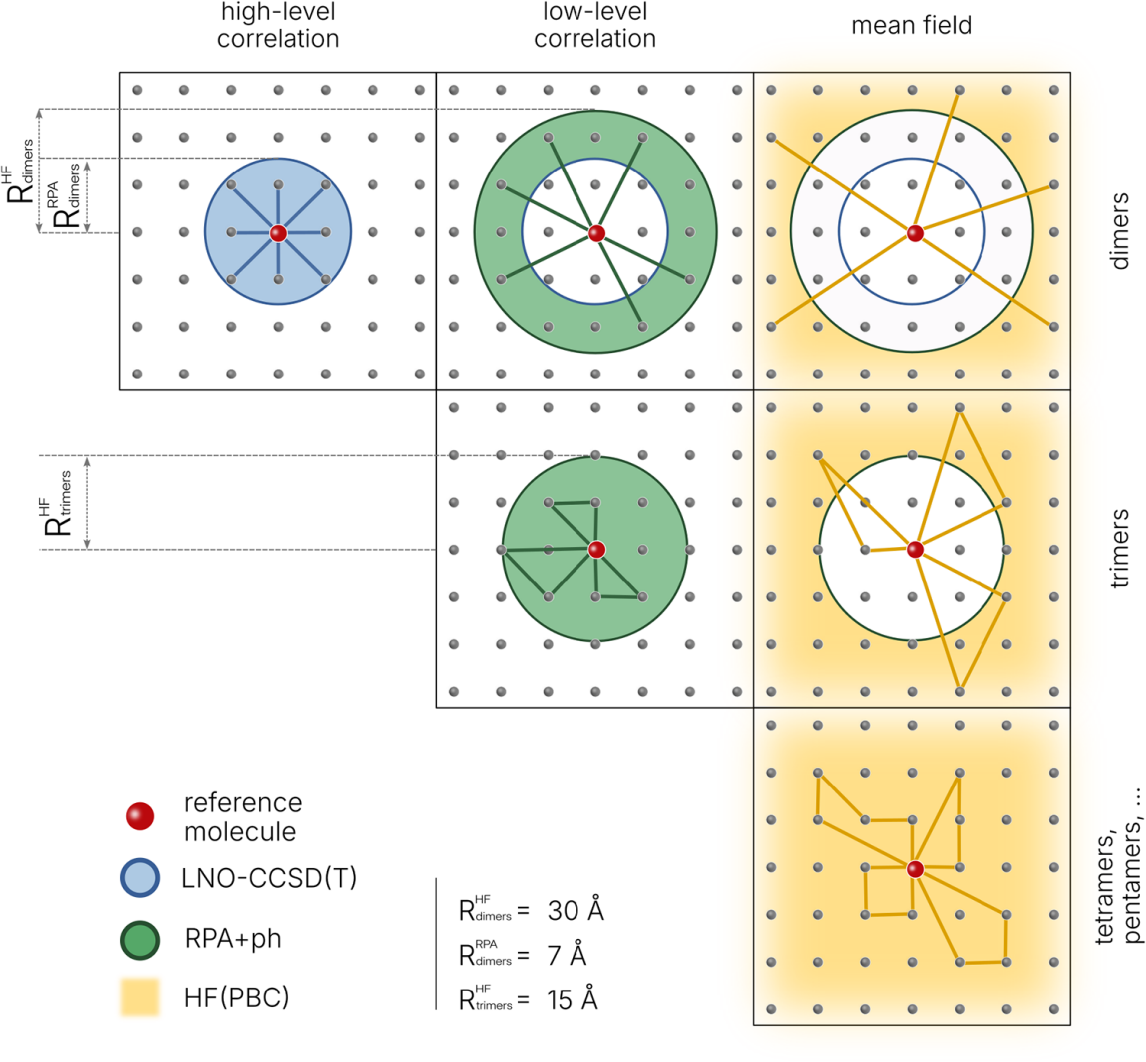
Partitioning of dimer, trimer, and higher-body contributions to the lattice energy into subsets treated with LNO–CCSD­(T), RPA+ph, and periodic HF, based on cluster type and intermolecular distance. A transition between levels of theory occurs if any pair of molecules within a cluster is separated by more than the switchover radius.

As the high-level model of electronic structure, we employ the LNO–CCSD­(T) method,^50–52^ which is a local coupled-cluster approximation with proven performance for noncovalent interaction energies.^53^ The high-level approach is limited to the monomer relaxation and dimer interaction energies below the switchover distance

5 $$R<R_{\mathrm{dimers}}^{\mathrm{RPA}}=7\;Å$$

This value of *R*_dimers_^RPA^ is suggested for wave function approaches in ref 36 and corresponds to, roughly, the distance of the second nearest-neighbors shell. Further extension of the high-level region has a diminishing effect on the accuracy, as we show in the results section.

The low-level description of electron correlation encompasses long-distance dimers with

6 $$R_{\mathrm{dimers}}^{\mathrm{RPA}}\leq R<R_{\mathrm{dimers}}^{\mathrm{HF}}=30\;Å$$

and all nonadditive trimer energies within the cutoff radius of

7 $$R<R_{\mathrm{trimers}}^{\mathrm{HF}}=15\;Å$$

In the data set considered in this work, the cutoffs defined in eqs 6 and 7 correspond to a few hundred symmetry-unique dimers and roughly ten thousand trimers, as shown in refs 36, 37 . The electronic structure model applied in the low-level region is RPA with beyond-RPA corrections. Even though this approach is more efficient than conventional coupled-cluster methodology, further extension of *R*_dimers_^HF^ and *R*_trimers_^HF^ is prohibitively expensive.

In polar molecules, extremely long-range interactions might occur due to polarization by static multipoles, for example, as in trimers of formamide at *R* = 20 Å.^37^ However, the existing results for ice^28^ and methanol^29^ indicate that those poorly convergent contributions are, essentially, mean-field effects^54^ and can be effectively captured by the energy difference

8 ΔHF(PBC)=HF(PBC)−HF(MBE)

where HF­(PBC) is the periodic HF total lattice energy and HF­(MBE) is a truncated MBE with monomer, dimer, and trimer contributions. Therefore, to extend the applicability of the multi-level approach to polar systems, we supplement the computational protocol with a plane-wave HF calculation of the molecular solid and include ΔHF­(PBC) in the total lattice energy. This formally introduces interactions up to infinite distance and all many-body nonadditive energies beyond trimers, albeit without electron correlation.

### 2.2 Low-Level Correlation: RPA with Corrections

We present the essential details of the low-level coupled-cluster approach, referred to as the beyond-RPA method, based on ref 47 and applied here with modifications related to the treatment of the antisymmetrized Coulomb interactions. The beyond-RPA approximation follows from the definition of the total electronic energy

9 $$E=E^{\mathrm{HF}}+E_{c}$$

where *E* ^HF^ is the self-consistent Hartree–Fock energy and the exact correlation energy, *E*_c_, is formulated as the coupled-cluster expectation value of the normal-ordered Hamiltonian *H*_N_

$$E_{c}=\frac{\langle e^{T}\Psi_{0}|H_{N}\left|e^{T}\Psi_{0}\right\rangle }{\langle e^{T}\Psi_{0}\left|e^{T}\Psi_{0}\right\rangle }$$ 10

The correlated wave function is parametrized by the cluster excitation operator, *T*, acting on the Hartree–Fock state Ψ_0_. By the linked-cluster theorem^33,55^

11 $$E_{c}=\sum_{l=0}^{\infty }\sum_{k=0}^{\infty }\frac{1}{l!k!}\left\langle \Psi_{0}\right|{\left\{{\left(T^{†}\right)}^{l}H_{N}T^{k}\right\}}_{C}\left|\Psi_{0}\right\rangle$$

where the curly braces with “C” denote a sum over all fully contracted terms connected to *H*_N_.

For a given source of the amplitudes, *T*, a truncation of the rhs of eq 11 defines an approximation of the correlation energy. To proceed, assume that (i) the amplitudes in eq 11 are restricted to double excitations and originate from the direct-ring coupled-cluster doubles equation,^46^ (ii) the direct-ring terms are summed up to infinity, (iii) the remaining non-ring contributions are retained only through the terms quadratic in *T*. These conditions yield^47^

$$E_{c}=E_{c}^{\mathrm{RPA}}+E_{c}^{\mathrm{ph}}+E_{c}^{\mathrm{pp}/\mathrm{hh}}+4^{\mathrm{th}}\;\mathrm{and}\;\mathrm{higher‐order}\;\mathrm{terms}$$ 12

where the terms on the rhs are, respectively, the conventional RPA correlation energy, the particle-hole (ph) corrections, and the particle–particle/hole–hole (pp/hh) corrections defined as

13 $$E_{c}^{\mathrm{ph}}=E_{c}^{\mathrm{SOSEX}}+E_{c}^{2b}+E_{c}^{2d}+E_{c}^{2g}+E_{c}^{2h}+2E_{c}^{2i}$$

and

14 $$E_{c}^{\mathrm{pp}/\mathrm{hh}}=E_{c}^{2e}+E_{c}^{2f}+E_{c}^{2k}+E_{c}^{2l}$$

Detailed expressions for these terms are provided in Table 1.

**Table 1:** Contributions in the Expansion of the Beyond-RPA Correlation Energy up to Terms Quadratic in the RPA Double Excitation Amplitudes *T* ^a^

ph contributions	pp/hh contributions
*E*_c_^RPA^ = 2∑_*aibj*_^orb^ (*ai*|*bj*) *T*_*ij*_^*ab*^ *E*_c_^SOSEX^ = −∑_*aibj*_^*orb*^ (*ai*|*bj*) *T*_*ji*_^*ab*^	*E*_c_^2*e*^ = 2∑_aibjkl_^orb^ (*ij*|*kl*) *T*_*ik*_^*ab*^ *T*_*lj*_^*ba*^
*E*_c_^2*b*^ = −4∑_*aibjck*_^orb^ (*ai*|*bj*) *T*_*ik*_^*ac*^ *T*_*jk*_^*cb*^ *E*_c_^2*d*^ = 2∑_*aibjck*_^orb^ (*ai*|*bj*) *T*_*ki*_^*ac*^ T_*jk*_^*cb*^	*E*_c_^2*f*^ = −∑_*aibjkl*_^orb^ (*ij*|*kl*)*T*_*ik*_^*ab*^ *T*_*jl*_^*ba*^
*E*_c_^2*g*^ = −4∑_*aibjck*_^orb^ (*ij*|*ab*) *T*_*jk*_^*ac*^ *T*_*ki*_^*cb*^ *E*_c_^2*h*^ = −4∑_*aibjck*_^orb^ (*ij*|*ab*) *T*_*kj*_^*ac*^ *T*_*ik*_^*cb*^	*E*_c_^2*k*^ = 2∑_*aibjcd*_^orb^ (*ab*|*cd*) *T*_*ij*_^*ac*^ *T*_*ji*_^*db*^
*E*_c_^2*i*^ = 2∑_*aibjck*_^orb^ (*ij*|*ab*) *T*_*jk*_^*ac*^ *T*_*ik*_^*cb*^	*E*_c_^2*l*^ = −∑_*aibjcd*_^orb^ (*ab*|*cd*) *T*_*ij*_^*ac*^ *T*_*ij*_^*db*^

a The terms are divided into the particle-hole (ph) and particle-particle/hole–hole (pp/hh) subsets according to the type of the Coulomb integral contracted with the 2-electron reduced density matrix (2-RDM).

Let us define various approximations which can be formed by the terms on the rhs of eq 12. The physical content of those approximations is summarized in Figure 2. The base RPA energy contains only the direct-ring contribution,

15 $$E^{\mathrm{RPA}}=E^{\mathrm{HF}}+E_{c}^{\mathrm{RPA}}$$

While efficient, *E*_c_^RPA^ lacks proper antisymmetry and introduces the exclusion-principle violating (EPV) terms, which propagate identical single-particle states at equal times.^56^ Corrections beyond *E* ^RPA^ should remove those unphysical terms.

**Figure 2:**
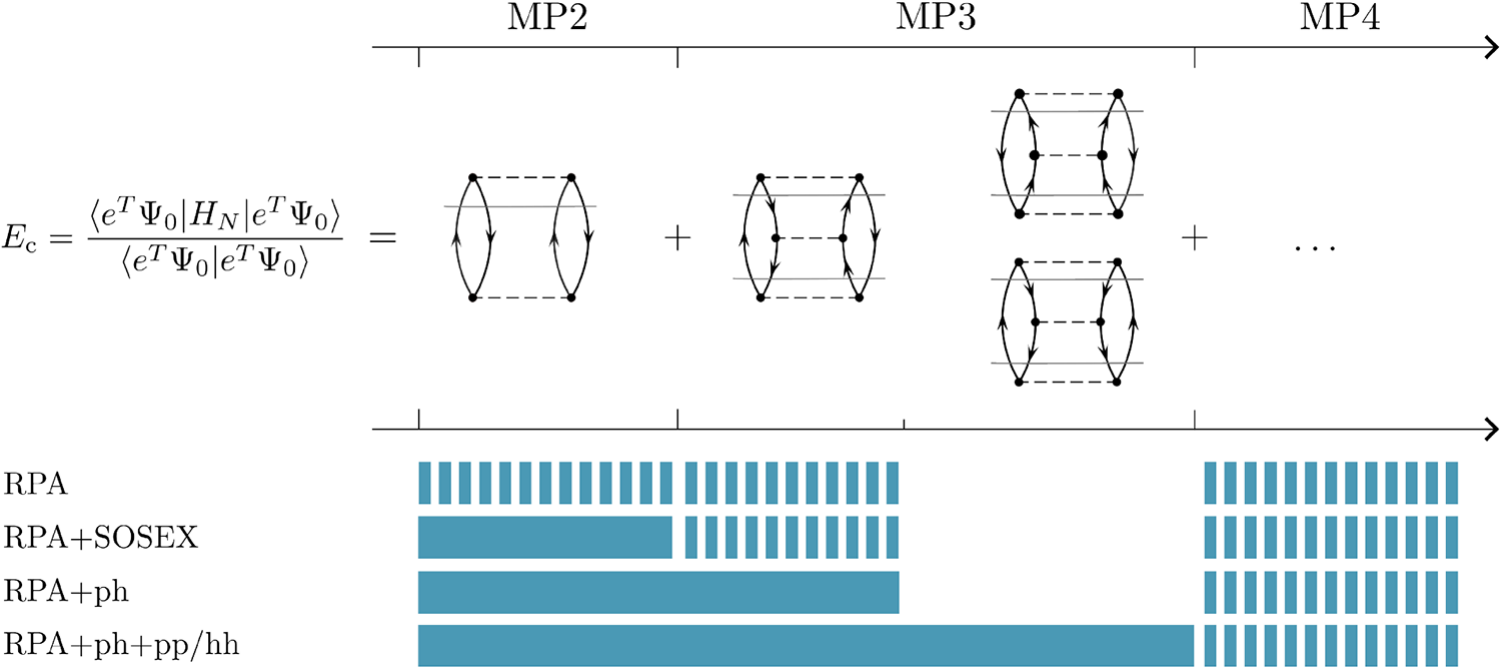
Perturbation theory expansion of RPA-based models of the correlation energy. Solid color corresponds to diagrams included with the exact prefactor at a given approximation level. Dashed colored bars correspond to incomplete and non-antisymmetric contributions. The convention of ref 33 is used: dashed lines represent antisymmetrized Coulomb integrals and solid horizontal lines are energy denominators.

The simplest approach which goes beyond RPA is the second-order screened exchange correction (SOSEX),^57,58^ where the energy

16 $$E^{\mathrm{RPA}+\mathrm{SOSEX}}=E^{\mathrm{HF}}+E_{c}^{\mathrm{RPA}}+E_{c}^{\mathrm{SOSEX}}$$

is free from the EPVs up to the second order of perturbation theory.^56,58^ Still, when applied with the self-consistent HF orbitals, RPA and RPA+SOSEX interaction energies differ only by a marginal amount.^49^

Third-order corrections are expected to have a much stronger impact on noncovalent interactions than SOSEX.^47,49,59^ Here, these corrections are introduced by *E*_c_^ph^ and *E*_c_^pp/hh^. In the most complete formulation

17 $$E^{\mathrm{RPA}+\mathrm{ph}+\mathrm{pp}/\mathrm{hh}}=E^{\mathrm{HF}}+E_{c}^{\mathrm{RPA}}+E_{c}^{\mathrm{ph}}+E_{c}^{\mathrm{pp}/\mathrm{hh}}$$

which reproduces the exact perturbation theory expansion through third order. However, a major drawback of this approximation is the high cost associated with Coulomb integrals involving four virtual indices in the *E*_c_^2*k*^ and *E*_c_^2*l*^ contributions from *E*_c_^pp/hh^. Thus, we will avoid this approach except for a few numerical tests which illustrate the magnitude of the pp/hh contribution in noncovalent systems.

A more practical approach balancing accuracy and cost retains only ph-type corrections

18 $$E^{\mathrm{RPA}+\mathrm{ph}}=E^{\mathrm{HF}}+E_{c}^{\mathrm{RPA}}+E_{c}^{\mathrm{ph}}$$

As illustrated in Figure 2, while *E* ^RPA+ph^ neglects the third-order pp and hh diagrams, it accounts for the ph diagram with its exact prefactor and proper antisymmetry.

In what follows, RPA+ph will serve as the lower-level correlated electronic structure method in the multi-level approach. This choice is supported by the numerical data shown in Section 2 of Supporting Information, which illustrate that the numerical differences between the efficient variant, RPA+ph, and the full variant, RPA+ph+pp/hh, are small and do not justify the extra computational cost.

### 2.3 Implementation Details

Due to the need to compute thousands of nonadditive trimer interactions, the low-level correlated step is best carried out with a highly customized implementation designed to balance efficiency and numerical stability—a fundamental challenge for MBE.^27^ Our beyond-rpa program^60^ achieves this goal by extensive use of tensor factorization techniques with tight numerical thresholds and automated estimation of the numerical error in the interaction energy.

The beyond-RPA post-SCF phase starts with evaluation of the RPA doubles amplitudes from the closed formula derived in ref 47. Two types of low-rank factorizations are carried out in succession. First, during the amplitude generation step, the amplitudes are computed from eq 38 of ref 47 by assembling the eigenvectors *U*_*ai*,μ_ and eigenvalues *a*_μ_ up to the cutoff threshold ε_EVD_

$$T_{\mathrm{ij}}^{\mathrm{ab}}=\underset{\mu }{\sum }U_{\mathrm{ai},\mu }a_{\mu }U_{\mathrm{bj},\mu }\;\mathrm{for}\;\left|a_{\mu }\right|>\varepsilon_{\mathrm{EVD}}=10^{-5}$$ 19

Computational cost is reduced since the number of significant eigenpairs scales linearly with system size for a given error tolerance.^61^

The second type of low-rank decomposition is a modified, SVD-based variant of the pair natural orbital (PNO) factorization presented in ref 62. The algorithm proceeds as follows. (i) First, the occupied indices *i* and *j* are transformed to the localized basis with the Boys-Foster procedure.^63,64^ (ii) The amplitude matrix, *T*_*ij*_^*ab*^, is aligned in memory as a quantity with free virtual indices *a* and *b* and fixed occupied indices *i* and *j*. (iii) The SVD rank reduction of such *N*_virt_ × *N*_virt_ matrix is executed up to the PNO threshold ε_PNO_. The resulting truncated expansion is

20 $$T_{\mathrm{ij}}^{\mathrm{ab}}=\sum_{x}P_{\mathrm{ax}}^{\left(\mathrm{ij}\right)}\sigma_{x}^{\left(\mathrm{ij}\right)}Q_{\mathrm{bx}}^{\left(\mathrm{ij}\right)}\;\mathrm{for}\;\sigma_{x}^{\left(\mathrm{ij}\right)}>\varepsilon_{\mathrm{PNO}}=10^{-6}$$

where *P* ^(*ij*)^ and *Q* ^(*ij*)^ are the singular vector matrices and σ_*x*_^(*ij*)^ is the *x*th singular value belonging to the occupied pair *ij*. For the systems discussed in the numerical section, we have verified that the number of significant PNOs depends weakly on the system size.

Once the PNOs are available, the ph beyond-RPA correction to the correlation energy is obtained as

21 $$E_{c}^{\mathrm{ph}}=E_{c}^{\mathrm{SOSEX}}+\sum_{\mathrm{aibj}}^{\mathrm{orb}}\left(\left(\mathrm{ai}|\mathrm{bj}\right)G_{\mathrm{aibj}}^{\prime }+\left(\mathrm{ij}|\mathrm{ab}\right)G_{\mathrm{ijab}}\right)$$

where two kinds of reduced density-like intermediates are used: (i) *G*_*ijab*_ gathers the contractions present in *E*_c_^2*g*^ + *E*_c_^2*h*^ + 2 *E*_c_^2*i*^

$$G_{\mathrm{ijab}}=\overset{\mathrm{orb}}{\underset{k}{\sum }}\underset{\mathrm{xy}}{\sum }\left(-4P_{\mathrm{ax}}^{\left(\mathrm{jk}\right)}S_{\mathrm{xy}}^{\left(\mathrm{jk},ki\right)}{\mathrm{Q̃}}_{\mathrm{by}}^{\left(\mathrm{ki}\right)}-4P_{\mathrm{ax}}^{\left(\mathrm{kj}\right)}S_{\mathrm{xy}}^{\left(\mathrm{kj},ik\right)}{\mathrm{Q̃}}_{\mathrm{by}}^{\left(\mathrm{ik}\right)}+4P_{\mathrm{ax}}^{\left(\mathrm{jk}\right)}S_{\mathrm{xy}}^{\left(\mathrm{jk},ik\right)}{\mathrm{Q̃}}_{\mathrm{by}}^{\left(\mathrm{ik}\right)}\right)$$ 22

whereas (ii) *G*_*aibj*_^′^ accounts for *E*_c_^2*b*^ + *E*_c_^2*d*^

$$G_{\mathrm{aibj}}^{\prime }=\overset{\mathrm{orb}}{\underset{k}{\sum }}\underset{\mathrm{xy}}{\sum }\left(-4P_{\mathrm{ax}}^{\left(\mathrm{ik}\right)}S_{\mathrm{xy}}^{\left(\mathrm{ik},jk\right)}{\mathrm{Q̃}}_{\mathrm{by}}^{\left(\mathrm{jk}\right)}+2P_{\mathrm{ax}}^{\left(\mathrm{ki}\right)}S_{\mathrm{xy}}^{\left(\mathrm{ki},jk\right)}{\mathrm{Q̃}}_{\mathrm{by}}^{\left(\mathrm{jk}\right)}\right)$$ 23

The intermediates in eqs 22 and 23 are the overlap matrix between PNOs

24 $$S_{\mathrm{xy}}^{\left(\mathrm{ij},i\prime j\prime \right)}=\sum_{a}^{\mathrm{orb}}{\mathrm{Q̃}}_{\mathrm{ax}}^{\left(\mathrm{ij}\right)}P_{\mathrm{ay}}^{\left(i\prime j\prime \right)}$$

and rescaled right singular vectors

25 $${\mathrm{Q̃}}_{\mathrm{ax}}^{\left(\mathrm{ij}\right)}=\sigma_{x}^{\left(\mathrm{ij}\right)}Q_{\mathrm{ax}}^{\left(\mathrm{ij}\right)}$$

The indices *x* and *y* traverse the PNO sets of the respective occupied pairs. The formulation given in eqs 21–24 reduces to dot products and matrix multiplications. Consequently, computing *E*_c_^ph^ achieves near-perfect utilization of modern hardware, which is highly optimized for basic linear algebra operations.

A unique feature of the RPA-based formalism is that numerical errors from the low-rank decomposition of *T* can be estimated at a negligible cost. As we have verified, a good estimate of the numerical error in the total correlation energy is the error in the inexpensive *E*_c_^RPA^ component. This error estimate is determined by recomputing *E*_c_^RPA^ in two ways: (i) exactly, using the density response function and integration over frequencies^65^ and (ii) approximately, using *T*-dependent formula for *E*_c_^RPA^ from Table 1 and decomposed amplitudes. The difference between these values provides an error estimate for interaction energies. The extra cost is negligible because the intermediates required for the accurate *E*_c_^RPA^ are already available during the computation of *T*.

## 3 Results

### 3.1 Computational Details

The multi-level approach involves three kinds of electronic single-point computations performed with different computer software: (i) LNO–CCSD­(T) for the monomers and short-range dimers, (ii) RPA with corrections for the long-range dimers and all trimers, (iii) and HF with periodic boundary conditions for the remaining physical contributions. The LNO–CCSD­(T) calculations were performed using the MRCC program,^66^ version from August 28, 2023, with the tightest available numerical settings (vvTight) unless noted otherwise. The RPA and beyond-RPA energies were evaluated using the beyond-rpa program developed as a part of this work and available at GitHub.^60^ Molecular cluster extraction from crystal supercells and input preparation for MBE calculations were performed using our customized Python script, mbe-automation,^60^ which integrates components from the open source programs ase,^67^ crystalatte,^68^ and membed.^31^

All RPA-based correlation energy expressions were evaluated using HF orbitals and orbital energies. The HF self-consistent field calculations which precede the RPA step were accelerated by the THC decomposition of the Coulomb integrals.^69,70^ THC-related numerical errors were eliminated perturbatively by a single post-SCF re-evaluation of the Fock Hamiltonian with the exact integrals. Both LNO–CCSD­(T) and RPA employed Duning’s triple- and quadruple-ζ correlation-consistent basis sets augmented with diffuse functions^71^ (see details in Section 1 of Supporting Information). All dimer and trimer energies were counterpoise-corrected with ghost atoms placed within each molecular cluster. The periodic HF calculations were carried out with VASP^72–74^ using the projector augmented wave method^75,76^ (PAW) with “standard” data sets and the plane-wave energy cutoff at 1100 eV. We applied the Coulomb cutoff technique^77^ (HFRCUT keyword in VASP), along with a dense *k*-point sampling for crystals and large cell sizes for molecules to achieve converged energy values without explicit extrapolation.^78^ Further details necessary to reproduce our calculations are summarized in Section 1 of Supporting Information.

### 3.2 Validation on the X23 Data Set

The X23 data set^15^ contains crystal structures of small, rigid molecules that vary in their dominant interaction types and total lattice energy values ranging from −29 kJ/mol for CO_2_ to −156 kJ/mol for cytosine.^24^ The systems are well-characterized both experimentally and computationally, with available reference sublimation enthalpies,^15,24,79^ total lattice energies,^24^ and isolated two- and three-body contributions.^36,40^ The best theoretical estimates of the X23 data, that is, the DMC values of ref 24, are consistent with the experiment within the experimental error range.^24^

#### 3.2.1 Two-Body Contributions

Pairwise interactions dominate the lattice energy (Figure 3), making it essential to control uncertainties in the two-body contribution: (i) errors from switching between LNO–CCSD­(T) and RPA+ph, (ii) LNO approximation errors,^53^ and (iii) incomplete convergence with distance. Our goal is to keep these uncertainties below 1 kJ/mol.

**Figure 3:**
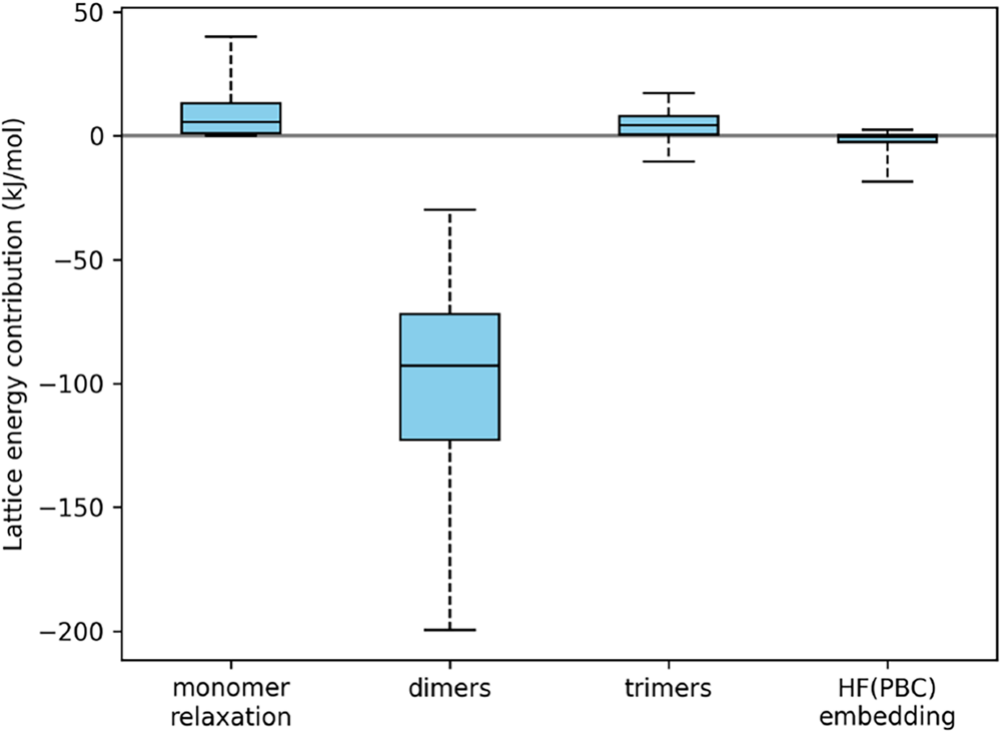
Overview of the MBE contributions to the crystal lattice energy in the entire X23 data set. The energies are obtained using the multi-level approach. The boxes extend from the first to the third quartile, and the whiskers extend from the minimum to the maximum energy. The HF­(PBC) embedding accounts for the sum of extremely long-distance contributions and the nonadditive terms beyond trimers.

A subset of ten X23 systems was selected to compare multi-level two-body lattice energies against canonical CCSD­(T) data of Sargent et al.^36^ Figure 4 shows how errors vary with the switchover distance and the choice of low-level correlation. In the case where only the low-level method is used for all distances, conventional RPA errors range from 7 kJ/mol (ammonia) to over 30 kJ/mol (hexamine). The SOSEX correction is negligible, while the ph correction significantly improves results, reducing errors by a factor of 3. However, achieving the 1 kJ/mol target requires connected triples at short distances. The combination of canonical CCSD­(T) and RPA+ph with *R*_dimers_^RPA^ = 7 Å—the switchover distance in our multi-level approach—meets this accuracy target.

**Figure 4:**
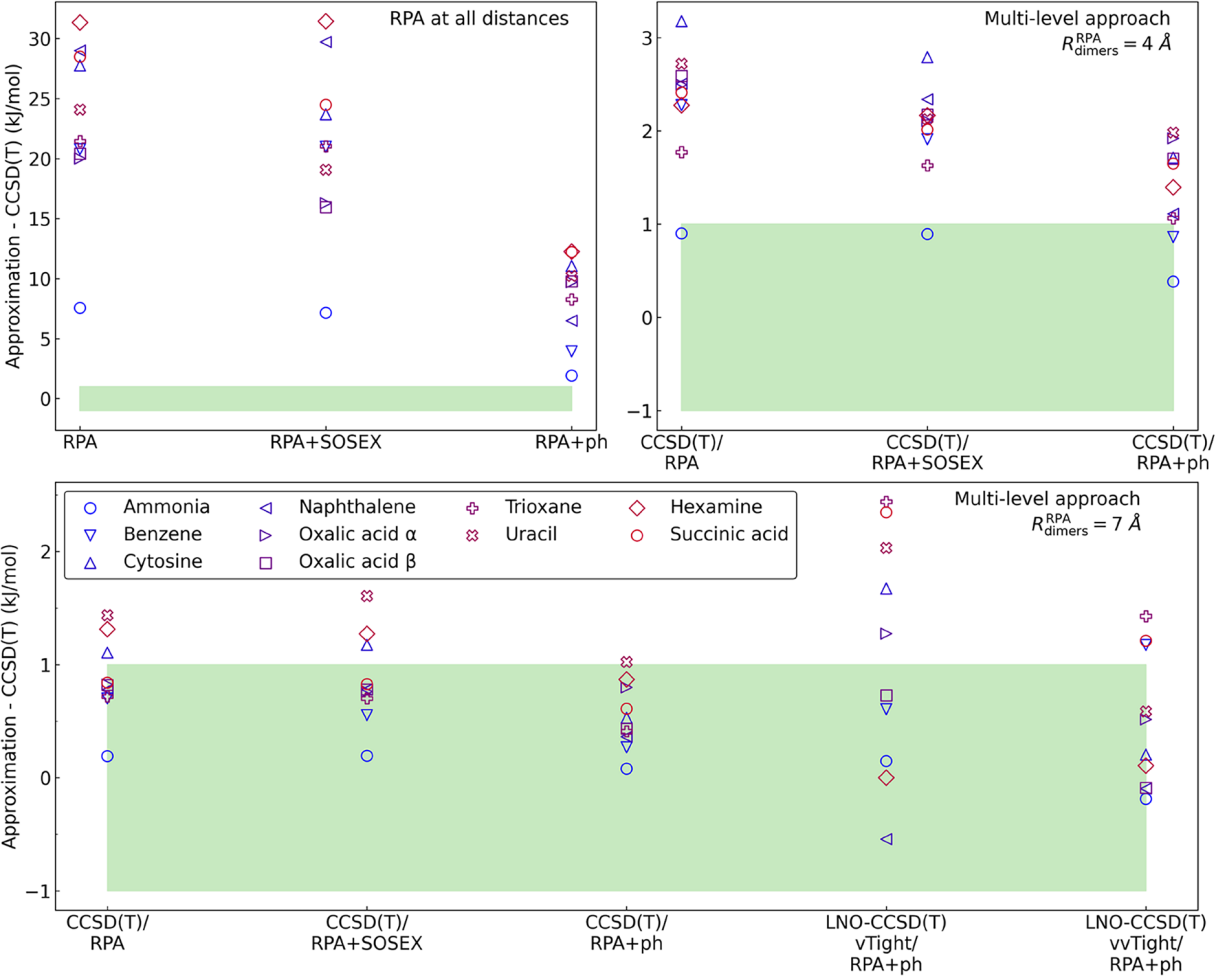
Dimer contribution to the crystal lattice energy for various choices of the switchover distance. *A*/*B* denotes a multi-level approach where method *A* is used for *R* < *R*_dimers_^RPA^ and *B* is applied for the remaining dimers at *R*_dimers_^RPA^ ≤ *R* < 30 Å. The canonical CCSD­(T) energies are taken from ref 36.

Substituting canonical CCSD­(T) for LNO–CCSD­(T) introduces additional, locality-related errors (Figure 4). For example, at the vTight level of accuracy settings,^52^ the numerical errors exceed 1 kJ/mol in succinic acid and trioxane. However, using the tightest available thresholds (vvTight), the approach adopted in our multi-level protocol, makes LNO–CCSD­(T)/RPA+ph practically equivalent to CCSD­(T)/RPA+ph.

The two-body errors due to incomplete distance convergence are negligible. As shown in Figure 5 and Section 5 of Supporting Information, the interaction energy curves plateau at 10 Å for dispersion-bonded systems and 15 Å for polar molecules, far before the *R*_dimers_^HF^ = 30 Å cutoff applied in our approach.

**Figure 5:**
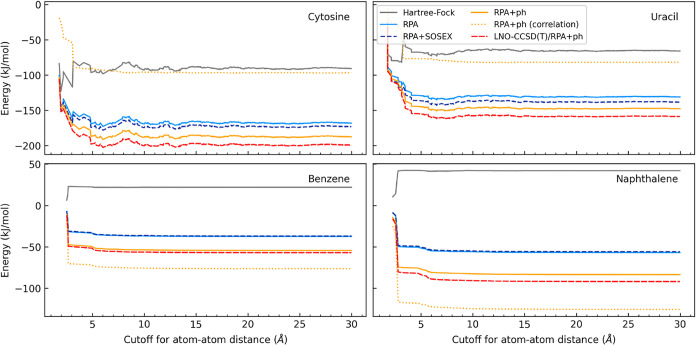
Two-body contribution to the crystal lattice energy as a function of the cutoff distance. The energies are accumulated up to a given cutoff radius, defined as the maximum atom–atom distance between molecules. The dotted line corresponds to the correlation energy component of the total RPA+ph energy.

#### 3.2.2 Three-Body Contributions

The nonadditive three-body energy is typically an order of magnitude smaller than the two-body term (Figure 3). Still, neglecting this contribution would lead to a significant error in the total energy and possibly an incorrect ordering of polymorphs. For example, in 1,4-cyclohexanedione, anthracene, cytosine, oxalic acid α, urea, and succinic acid the three-body term exceeds 10 kJ/mol at the RPA+ph level (Section 6 in Supporting Information). In oxalic acid, the three-body nonadditive energy changes sign between the α and β polymorphs, directly influencing their relative stability. In the context of the multi-level approach, this necessitates a sufficient treatment of electron correlation and tight convergence with the cutoff distance. A reasonable error tolerance is 1 kJ/mol and the correct sign in the cases with small three-body contributions.

Let us consider two systems from the X23 set for which the reference CBS-extrapolated canonical CCSD­(T) data are available: benzene^80^ and acetic acid.^37^ When applied for all distances, RPA underestimates the three-body term by 1.5 kJ/mol for benzene and 0.6 kJ/mol for acetic acid (Figure 6). Adding the SOSEX correction has a negligible effect. By contrast, the RPA+ph variant overestimates the three-body energies by only about 0.5 kJ/mol in both systems. This result provides a rough estimate of the three-body error in our protocol, where RPA+ph is applied for all trimers without any high-level corrections.

**Figure 6:**
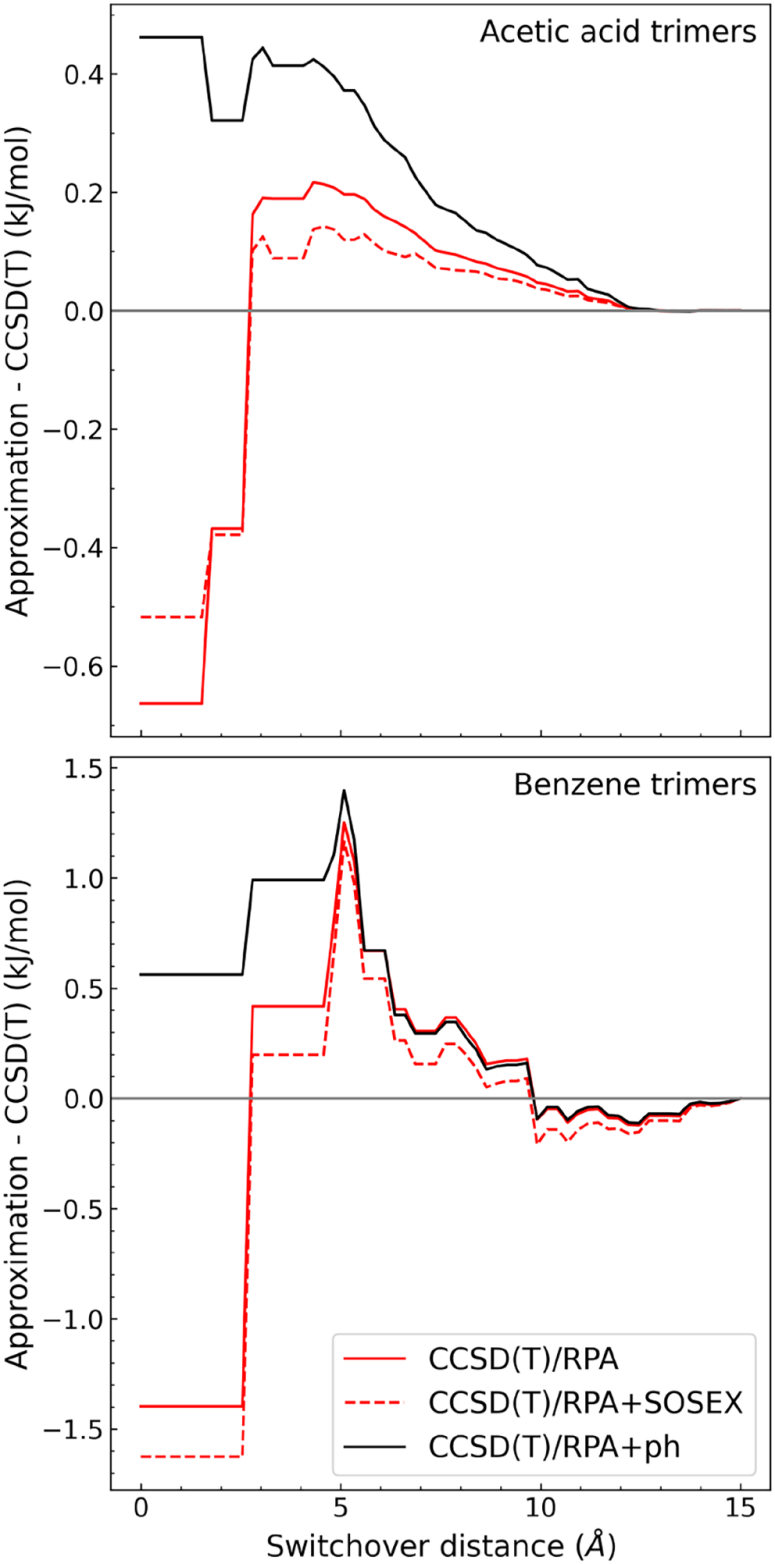
Error in the nonadditive three-body interaction energy as a function of the switchover distance between CCSD­(T) and RPA-based approximations. The CBS-extrapolated canonical CCSD­(T) energies are taken from ref 37 and 80. *A*/*B* denotes a multi-level approximation, where *A* is used below the switchover distance and *B* is applied for all remaining distances up to 15 Å.

An important source of three-body errors is an incomplete convergence with distance. As shown in Figure 7, three-body interactions in dispersion-bound systems (benzene, naphthalene) stabilize around 10 Å, whereas in polar systems (cytosine, uracil), significant fluctuations persist even at 15 Å. Capturing such long-distance contributions using MBE is computationally not feasible. However, an inspection of Figure 7 reveals that the correlation-only three-body energy in polar systems converges fast, at a rate comparable to that of the total energy curve in nonpolar systems. Thus, the poorly convergent long-range fluctuations reside in the Hartree–Fock term—supporting the inclusion of the periodic Hartree–Fock correction, ΔHF­(PBC), in the multi-level lattice energy.

**Figure 7:**
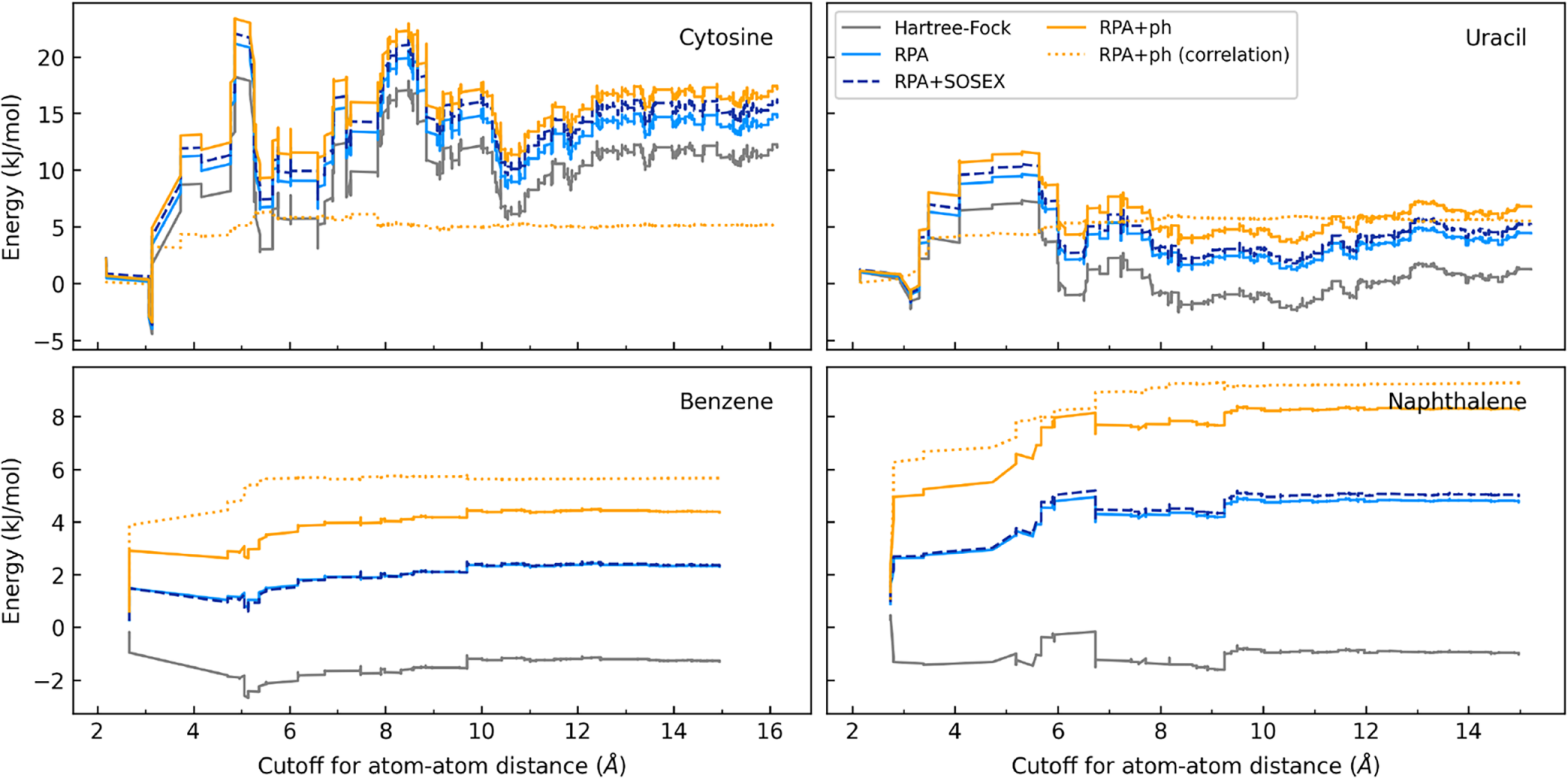
Nonadditive three-body contribution to the crystal lattice energy as a function of the cutoff distance. The energies are accumulated up to a given cutoff radius, defined as the maximum atom–atom distance between constituent molecules in a trimer. The dotted line corresponds to the correlation energy component of the total RPA+ph energy.

#### 3.2.3 Total Lattice Energy

The main result of this work is demonstrated in Figure 8, where, upon refinement of the theoretical model, the multi-level lattice energies systematically converge toward the reference DMC values within chemical accuracy of approximately ± 4 kJ/mol. This level of agreement with the reference is achieved in consecutive steps by the inclusion of (i) the RPA correlation, (ii) beyond-RPA corrections, (iii) LNO–CCSD­(T) for the monomer relaxation and short-distance dimers, and (iv) the HF­(PBC) embedding, each with a visible effect on the average and/or the spread of signed errors. At the RPA correlated level, with MBE encompassing monomers, dimers, and trimers, the total lattice energy is severely underestimated (mean absolute error, MAE = 22.0 kJ/mol). Adding the SOSEX correction has a negligible influence, slightly decreasing MAE to 20.6 kJ/mol. The inclusion of the ph beyond-RPA corrections decreases the average error to 6.9 kJ/mol, mostly due to improved two-body interactions, as shown in Section 3.2.1. The remaining underbinding error is eliminated upon addition of connected triple excitations in the nearest dimers. In the LNO–CCSD­(T)/RPA+ph multi-level approach, the errors are distributed evenly around zero with MAE = 3.5 kJ/mol and RMSE = 4.8 kJ/mol. Still, some outliers remain, such as trioxane, for which the energy is underestimated by 12.7 kJ/mol, likely due to the missing contributions from the four- and higher-body nonadditive interactions.

**Figure 8:**
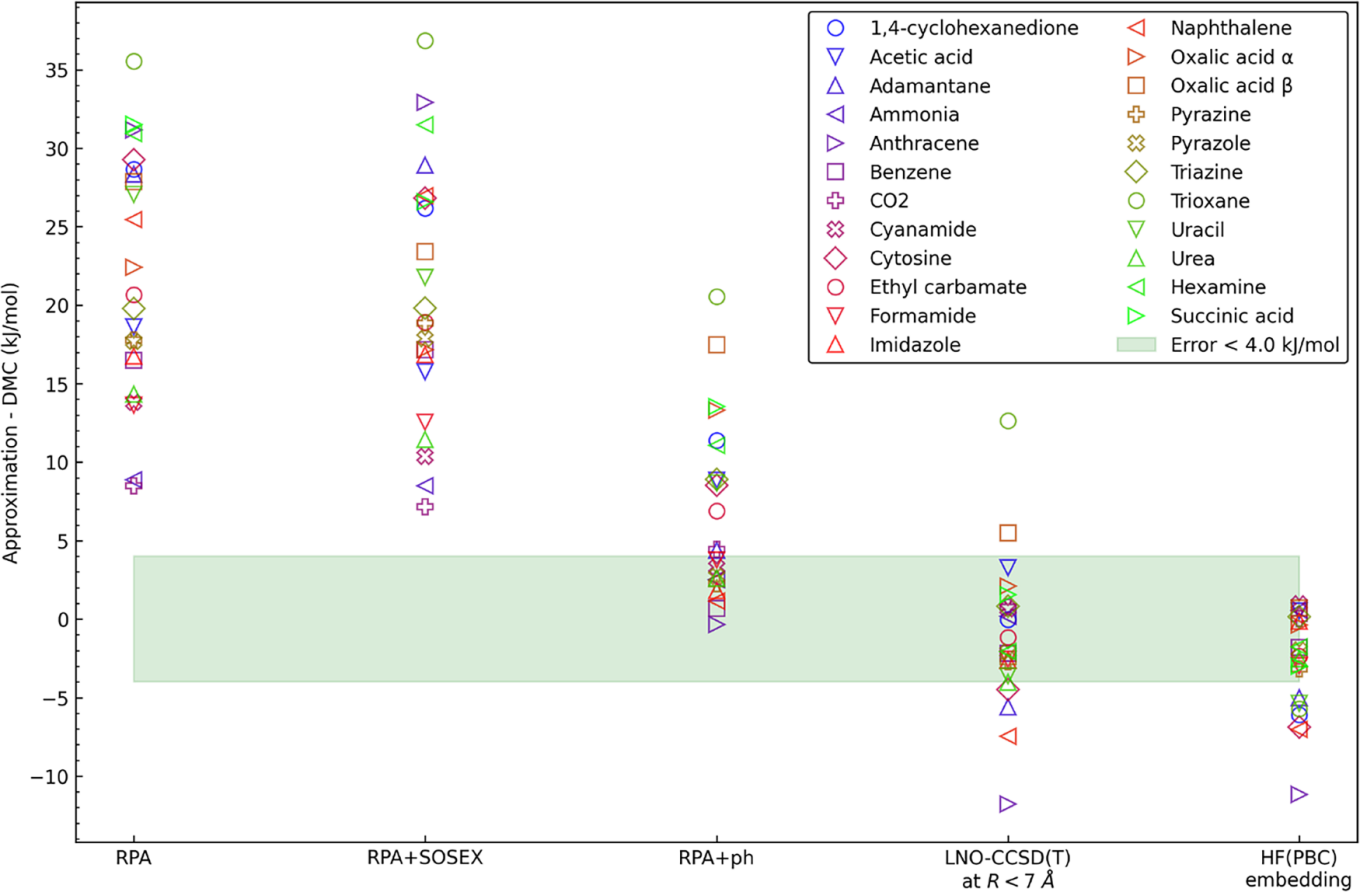
Convergence of the crystal lattice energy toward the accurate value in a series of electronic-structure approximations. The abscissa represents the features added to the multi-level approximation at each step. The HF­(PBC) embedding accounts for interactions at *R* ≥ *R* ^HF^ and *n* > 3-body terms. The signed errors are computed against the reference DMC energies from ref 24.

The final step in the multi-level protocol adds the periodic HF correction, which is an estimate of the physical contributions missing from the truncated MBE. The resulting multi-level lattice energies, presented in Table 2, show further improvement. The error measures for the complete protocol are MAE = 3.1 kJ/mol and RMSE = 4.2 kJ/mol. The signed errors indicate overbinding relative to the DMC reference, with two notable cases outside of the ±4 kJ/mol window: naphthalene and anthracene. Further investigation is needed to determine to what degree these errors stem from a systematic discrepancy between CCSD­(T) and DMC in large π–electron systems.^81,82^ Part of the error in the multi-level protocol arises from using standard PAW potentials^83,84^ in the HF­(PBC) contribution, likely affecting polar systems the most.^83,84^ A correction, such as the one defined in eq 1 of ref 83, should be applied to eliminate this error.

**Table 2:** Total Lattice Energies (kJ/mol) in the X23 Data Set

system	multi-level	DMC^24^
1,4-cyclohexanedione	–94.4	–88.3
acetic acid	–71.2	–71.7
adamantane	–66.0	–61.0
ammonia	–37.6	–38.2
anthracene	–111.4	–100.2
benzene	–51.6	–49.8
CO_2_	–29.4	–29.4
cyanamide	–82.6	–83.6
cytosine	–163.1	–156.2
ethyl carbamate	–86.5	–84.2
formamide	–84.0	–81.0
imidazole	–88.4	–88.2
naphthalene	–82.5	–75.5
oxalic acid α	–103.0	–102.6
oxalic acid β	–101.6	–102.3
pyrazine	–64.3	–61.1
pyrazole	–79.3	–77.3
triazine	–60.3	–60.5
trioxane	–67.8	–62.1
uracil	–139.7	–134.3
urea	–111.0	–108.5
hexamine	–88.0	–86.2
succinic acid	–128.2	–125.2
MAE	3.1	
RMSE	4.2	
MSE	–2.8	

## 4 Conclusions

We have presented a multi-level MBE approximation of the crystal lattice energy based on coupled-cluster wave function models: LNO–CCSD­(T) (high level) and RPA+ph (low level). The LNO–CCSD­(T) approximation is applied only for monomer relaxation and dimers at *R* < 7 Å, roughly within the first two coordination shells. This provides good accuracy for the dominant part of the lattice energy while limiting the expensive approach to a manageable number of subsystems. The remaining contribution from long-range dimers and trimers is treated with the RPA-based method—the simplest correlated wave function approximation which qualitatively represents many-body dispersion interactions. Conventional RPA is refined with beyond-RPA corrections, which mitigate the underestimation of pairwise interactions. Finally, the periodic Hartree–Fock correction is included to accelerate convergence to the bulk limit. Each component of the computational protocol systematically improves the accuracy of lattice energies for the X23 set of molecular solids. The final mean absolute error, 3.1 kJ/mol, is within the chemical accuracy target. This result supports the use of the beyond-RPA approach as a low-level correlated component in multi-level schemes based on coupled-cluster theory.

## Supplementary Material



## Notes

Additional plots and tables with raw numerical data; atomic coordinates, basis sets, and LNO coupled-cluster numerical settings applied in the multi-level calculations; details of the periodic Hartree-Fock calculations (PDF)
