# Supplementary material for: Multi-Level Coupled-Cluster Description of Crystal Lattice Energies
Source: J Chem Theory Comput. 2025 May 29;21(11):5533–44. doi: 10.1021/acs.jctc.5c00428 (PMC12159999; doi:10.1021/acs.jctc.5c00428)
Supplement: Supplementary file 1 [file ct5c00428_si_001.pdf]

Supporting Information: Multi-level coupled-cluster description of crystal lattice energies

Krystyna Syty,† Grzegorz Czekao,† Khanh Ngoc Pham,‡ and Marcin Modrzejewski*,†

†*University of Warsaw, Faculty of Chemistry, 02-093 Warsaw, Pasteura 1, Poland*

‡*Department of Chemical Physics and Optics, Faculty of Mathematics and Physics, Charles University, Ke Karlovu 3, CZ-12116 Prague 2, Czech Republic*

E-mail: m.m.modrzejewski@gmail.com

Contents

1 Technical details **S-3**

2 Tests of the low-level correlation variants **S-5**

3 Individual MBE contributions in the X23 data set **S-6**

4 Multi-level lattice energies with alternative variants of RPA **S-9**

5 Cutoff-distance dependence of the MBE terms **S-10**

5.1 1,4-cyclohexanedione S-10

5.2 Acetic acid S-11

5.3 Adamantane S-12

5.4 Ammonia . . . S-13
5.5 Anthracene . . . S-14
5.6 Benzene . . . S-15
5.7 CO2 . . . S-16
5.8 Cyanamide . . . S-17
5.9 Cytosine . . . S-18
5.10 Ethyl carbamate . . . S-19
5.11 Formamide . . . S-20
5.12 Idazole . . . S-21
5.13 Naphthalene . . . S-22
5.14 Oxalic acid α . . . S-23
5.15 Oxalic acid β . . . S-24
5.16 Pyrazine . . . S-25
5.17 Pyrazole . . . S-26
5.18 Triazine . . . S-27
5.19 Trioxane . . . S-28
5.20 Uracil . . . S-29
5.21 Urea . . . S-30
5.22 Hexamethylenetetramine . . . S-31
5.23 Succinic acid . . . S-32

References **S-32**

1 Technical details

Table S1: Atomic coordinates, basis sets, and LNO coupled-cluster numerical settings applied in the multi-level calculations.

System	Coordinates[d]		Basis set[a]	LNO-CC[b]
Section 2 of Supporting Information				
$CH_4 \cdots (H_2O)_{20}$	S1,S2	dimers	AVTZ→AVQZ	
		trimers	AVTZ→AVQZ	
Acetylene I, II	S3	dimers	AVTZ→AVQZ	
		trimers	AVTZ	
Section 3.2.1 Two-body contributions				
Ammonia, benzene, cytosine, naphthalene, oxalic acid α, oxalic acid β, trioxane, uracil, hexamine, succinic acid	S4,S5	dimers	AVTZ→AVQZ	vvTight and vTight
Section 3.2.2 Three-body contributions				
Acetic acid, benzene	S5,S6	trimers	AVTZ	
Section 3.2.3 Total lattice energy				
All systems in X23	S7	monomers	AVTZ→AVQZ	vvTight
		dimers	AVTZ→AVQZ	vvTight[c]
		trimers	AVTZ	
		periodic HF	plane waves, cutoff 1100 eV	

[a] Labels of the aug-cc-pVTZ and aug-cc-pVQZ correlation-consistent basis sets with diffuse functions[S8] are abbreviated as AVTZ and AVQZ, respectively. The right arrow denotes interaction energies extrapolated using the scheme of Halkier et al.[S9] In monomers and dimers, the RPA and LNO-CCSD(T) calculations employ identical basis sets. All dimers and trimers treated with MBE include the basis-set counterpoise correction with ghost atoms placed within each molecular cluster.
[b] Numerical accuracy settings for LNO-CCSD(T) applied in the MRCC program, version from August 28, 2023.
[c] Except for the anthracene monomers and dimers, where the vTight settings were used.
[d] Atomic coordinates from the literature were used without re-optimization.

Table S2: Details of the periodic Hartree-Fock calculations. The lattice energies were obtained using the formula $E_{\mathrm{latt}}^{\mathrm{HF}} = E_{\mathrm{crystal}}^{\mathrm{HF}}/N - E_{\mathrm{molecule}}^{\mathrm{HF}}$, where N is number of molecules in a unit cell. The energies of isolated molecules, $E_{\mathrm{molecule}}^{\mathrm{HF}}$, were computed using a simulation box of at least 21 Å in each dimension.

System	k-point grid
1,4-cyclohexanedione	$4 \times 4 \times 4$
Acetic acid	$3 \times 9 \times 6$
Adamantane	$4 \times 4 \times 4$
Amonia	$4 \times 4 \times 4$
Anthracene	$5 \times 5 \times 5$
Benzene	$4 \times 4 \times 4$
CO_2	$4 \times 4 \times 4$
Cyanamide	$4 \times 4 \times 4$
Cytosine	$3 \times 6 \times 9$
Ethyl carbamate	$4 \times 4 \times 4$
Formamide	$8 \times 4 \times 6$
Imidazole	$5 \times 5 \times 5$
Naphthalene	$4 \times 4 \times 4$
Oxalic acid α	$4 \times 4 \times 4$
Oxalic acid β	$5 \times 5 \times 5$
Pyrazine	$4 \times 6 \times 8$
Pyrazole	$5 \times 3 \times 7$
Triazine	$4 \times 5 \times 6$
Trioxane	$4 \times 4 \times 4$
Uracil	$3 \times 3 \times 9$
Urea	$4 \times 4 \times 4$
Hexamine	$4 \times 4 \times 4$
Succinic acid	$3 \times 3 \times 3$

2 Tests of the low-level correlation variants

Table S3: Pairwise and nonadditive energy contributions (kJ/mol) in $CH_4@(H_2O)_{20}$,[a] acetylene I (orthorhombic),[b] and acetylene II (cubic).[b]

	$CH_4@(H_2O)_{20}$		Acetylene I		Acetylene II	
	dimers[c]	trimers[d]	dimers	trimers	dimers	trimers
RPA	-12.5	2.2	-21.6	0.6	-20.7	0.9
RPA+SOSEX	-12.5	2.3	-21.8	0.7	-21.1	1.0
RPA+ph	-22.0	4.1	-28.5	1.6	-26.5	1.6
RPA+ph+pp/hh	-20.3	3.7	-27.7	1.4	-26.0	1.5
CCSD	-19.6	3.5	-22.4	0.7	-21.6	1.0
CCSD(T)	-26.4	4.3	-27.6	1.0	-25.9	1.2

[a] Endohedral water cage cluster[S1] Reference energies from ref S2.
[b] Distance cutoffs for the pairwise and nonadditive interaction energies in acetylene are defined as in ref S3. Reference energies are taken from ref S3.
[c] Sum of 20 pairwise interaction energies of $CH_4 \cdots H_2O$ dimers.
[d] Sum of 190 nonadditive interaction energies of $CH_4 \cdots H_2O \cdots H_2O$ trimers.

To assess the performance of different low-level correlation variants from Section 2.2, we examine representative many-body noncovalent systems: a methane molecule in a dodecahedral water cage[S2] and two forms of the acetylene molecular crystal.[S10] As shown in Table S3, RPA without corrections qualitatively captures three-body effects but severely underestimates two-body interactions. For instance, the RPA binding energy of $CH_4 \cdots H_2O$ dimers is too shallow, lying about 7 kJ/mol above CCSD, which itself underestimates the CCSD(T) reference by another 7 kJ/mol. Including the particle-hole correction E_c^{ph} improves two-body interactions while maintaining the accuracy of three-body terms. Although the binding energy of $CH_4 \cdots (H_2O)_{20}$ remains underestimated, the error is within the expected range for a doubles-only approach.[S2] Going beyond RPA+ph by adding $E_c^{pp/hh}$ has only a minor impact, contributing about 2 kJ/mol in $CH_4 \cdots (H_2O)_{20}$ and less than 1 kJ/mol in acetylene. Since further corrections provide little improvement at a significant computational cost, we adopt RPA+ph as the low-level correlated method in our multi-level approach alongside LNO-CCSD(T).

3 Individual MBE contributions in the X23 data set

Table S4: Monomer relaxation energies, dimer interaction energies, and nonadditive trimer interaction energies contributing to the total lattice energies in the X23 data set at various levels of approximations. The RPA and RPA+ph labels denote pure RPA-based energies of dimers with $R < R^{\mathrm{HF}}_{\mathrm{dimers}}$ and trimers with $R < R^{\mathrm{HF}}_{\mathrm{trimers}}$. ΔLNO-CC is the correction defined as LNO-CCSD(T) − RPA+ph and applied to the monomers and dimers with $R < R^{\mathrm{RPA}}_{\mathrm{dimers}}$. Σ denotes the sum of the RPA+ph energy and the ΔLNO-CC correction.

System		RPA	RPA+ph	ΔLNO-CC	Σ
1,4-cyclohexanedione	monomers	6.3	4.5	-0.8	3.7
	dimers	-74.8	-92.2	-10.6	-102.9
	trimers	8.9	10.8		10.8
Acetic acid	monomers	14.6	12.7	-0.4	12.2
	dimers	-66.9	-76.2	-5.1	-81.4
	trimers	-0.7	0.7		0.7
Adamantane	monomers	2.4	1.4	-0.3	1.1
	dimers	-37.1	-62.2	-9.7	-71.9
	trimers	2.0	4.2		4.2
Ammonia	monomers	1.9	1.2	-0.1	1.1
	dimers	-30.9	-37.1	-2.2	-39.4
	trimers	-0.4	0.2		0.2
Anthracene	monomers	3.1	1.4	-0.5	0.9
	dimers	-78.7	-113.1	-11.0	-124.1
	trimers	6.6	11.2		11.2
Benzene	monomers	1.7	0.8	-0.2	0.6
	dimers	-37.3	-54.3	-2.7	-57.0
	trimers	2.3	4.4		4.4
CO_2	monomers	-0.1	-0.1	0.0	-0.0
	dimers	-21.4	-26.1	-3.7	-29.8

System		RPA	RPA+ph	ΔLNO-CC	Σ
	trimers	0.6	1.2		1.2
Cyanamide	monomers	10.9	10.0	-0.0	9.9
	dimers	-79.2	-90.3	-2.6	-92.9
	trimers	-1.5	0.1		0.1
Cytosine	monomers	26.8	23.0	-1.3	21.7
	dimers	-168.4	-187.8	-11.8	-199.6
	trimers	14.7	17.2		17.2
Ethyl carbamate	monomers	9.5	7.9	-0.5	7.4
	dimers	-75.2	-88.8	-7.5	-96.3
	trimers	2.2	3.5		3.5
Formamide	monomers	13.6	11.0	-0.9	10.1
	dimers	-81.6	-89.2	-5.4	-94.6
	trimers	0.6	0.9		0.9
Imidazole	monomers	8.7	6.6	-0.1	6.5
	dimers	-74.1	-88.6	-4.3	-93.0
	trimers	-6.1	-4.3		-4.3
Naphthalene	monomers	2.0	0.9	-0.3	0.6
	dimers	-56.8	-83.5	-8.3	-91.8
	trimers	4.8	8.3		8.3
Oxalic acid α	monomers	39.5	39.2	-1.0	38.2
	dimers	-107.5	-118.2	-10.2	-128.4
	trimers	-12.2	-10.3		-10.3
Oxalic acid β	monomers	43.5	41.5	-1.5	40.0
	dimers	-122.9	-133.6	-10.5	-144.1
	trimers	4.9	7.3		7.3

System		RPA	RPA+ph	ΔLNO-CC	Σ
Pyrazine	monomers	3.0	1.6	-0.4	1.2
	dimers	-51.1	-67.2	-4.5	-71.7
	trimers	4.7	6.7		6.7
Pyrazole	monomers	7.5	5.7	-0.2	5.5
	dimers	-65.3	-80.1	-4.7	-84.7
	trimers	-1.8	-0.2		-0.2
Triazine	monomers	3.3	1.8	-0.4	1.4
	dimers	-47.7	-58.9	-7.7	-66.6
	trimers	3.7	5.6		5.6
Trioxane	monomers	7.0	4.7	-0.9	3.8
	dimers	-40.7	-54.8	-7.0	-61.8
	trimers	7.1	8.5		8.5
Uracil	monomers	19.5	15.4	-1.3	14.2
	dimers	-131.1	-147.8	-11.0	-158.9
	trimers	4.4	6.8		6.8
Urea	monomers	21.7	19.8	-0.3	19.5
	dimers	-105.3	-115.4	-6.3	-121.7
	trimers	-10.6	-10.3		-10.3
Hexamethylenetetramine	monomers	-0.1	0.2	0.0	0.2
	dimers	-58.9	-81.0	-13.1	-94.1
	trimers	3.7	5.7		5.7
Succinic acid	monomers	33.3	30.0	-0.5	29.5
	dimers	-134.9	-152.3	-11.5	-163.9
	trimers	8.0	10.7		10.7

4 Multi-level lattice energies with alternative variants of RPA

Table S5: Total lattice energies (kJ/mol) in the X23 data set obtained with alternative variants of the multi-level approximation based on RPA, RPA+SOSEX, and RPA+ph. The RPA-based low-level approximation of the correlation energy is applied in the long-distance dimers at $R^{\mathrm{RPA}}_{\mathrm{dimers}} \leq R < R^{\mathrm{HF}}_{\mathrm{dimers}}$ and in the trimers at $R < R^{\mathrm{HF}}_{\mathrm{trimers}}$. Each variant of the lattice energy contains the LNO-CCSD(T) and HF(PBC) contributions.

System	Multi-level			DMC[S7]
	RPA	RPA+SOSEX	RPA+ph	
1,4-cyclohexanedione	-95.9	-95.1	-94.4	-88.3
Acetic acid	-72.2	-71.9	-71.2	-71.7
Adamantane	-67.9	-67.8	-66.0	-61.0
Ammonia	-38.1	-38.2	-37.6	-38.2
Anthracene	-115.4	-115.0	-111.4	-100.2
Benzene	-53.3	-53.3	-51.6	-49.8
CO_2	-29.8	-29.7	-29.4	-29.4
Cyanamide	-84.0	-84.0	-82.6	-83.6
Cytosine	-165.1	-163.7	-163.1	-156.2
Ethyl carbamate	-87.7	-87.5	-86.5	-84.2
Formamide	-84.3	-83.6	-84.0	-81.0
Imidazole	-89.9	-89.9	-88.4	-88.2
Naphthalene	-85.6	-85.4	-82.5	-75.5
Oxalic acid α	-104.8	-105.1	-103.0	-102.6
Oxalic acid β	-103.8	-103.9	-101.6	-102.3
Pyrazine	-66.0	-65.9	-64.3	-61.1
Pyrazole	-80.6	-80.5	-79.3	-77.3
Triazine	-61.9	-61.9	-60.3	-60.5
Trioxane	-68.9	-68.7	-67.8	-62.1
Uracil	-141.5	-140.6	-139.7	-134.3
Urea	-110.8	-109.8	-111.0	-108.5
Hexamine	-89.5	-89.4	-88.0	-86.2
Succinic acid	-130.6	-130.3	-128.2	-125.2
MAE	4.4	4.1	3.1	
RMSE	5.7	5.4	4.2	
MSE	-4.4	-4.1	-2.8	

5 Cutoff-distance dependence of the MBE terms

5.1 1,4-cyclohexanedione

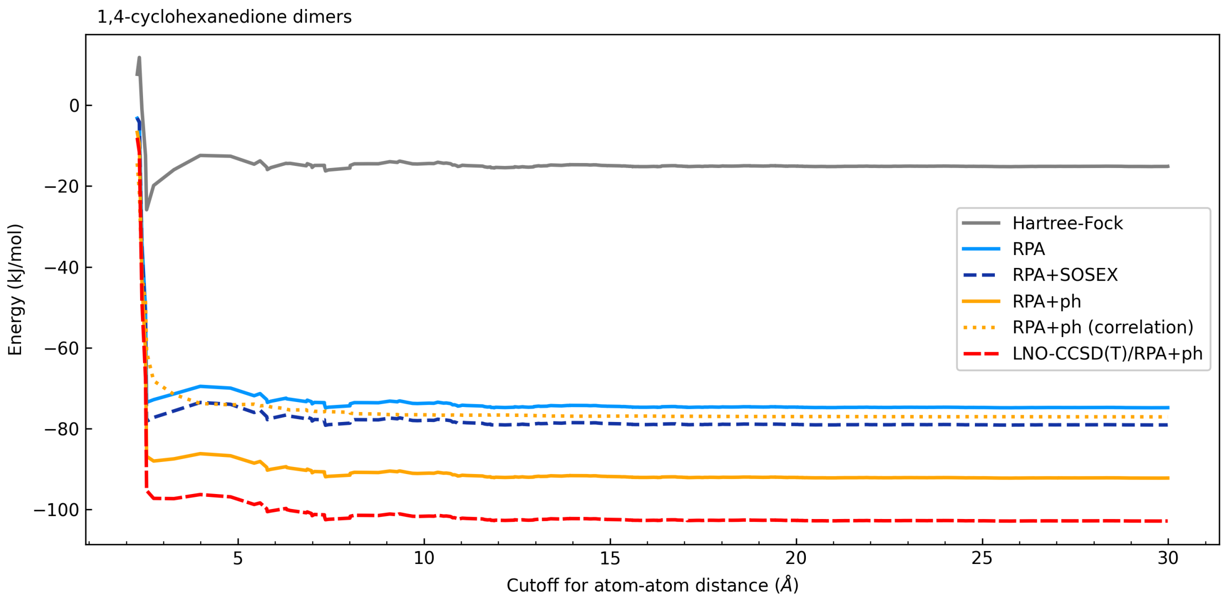

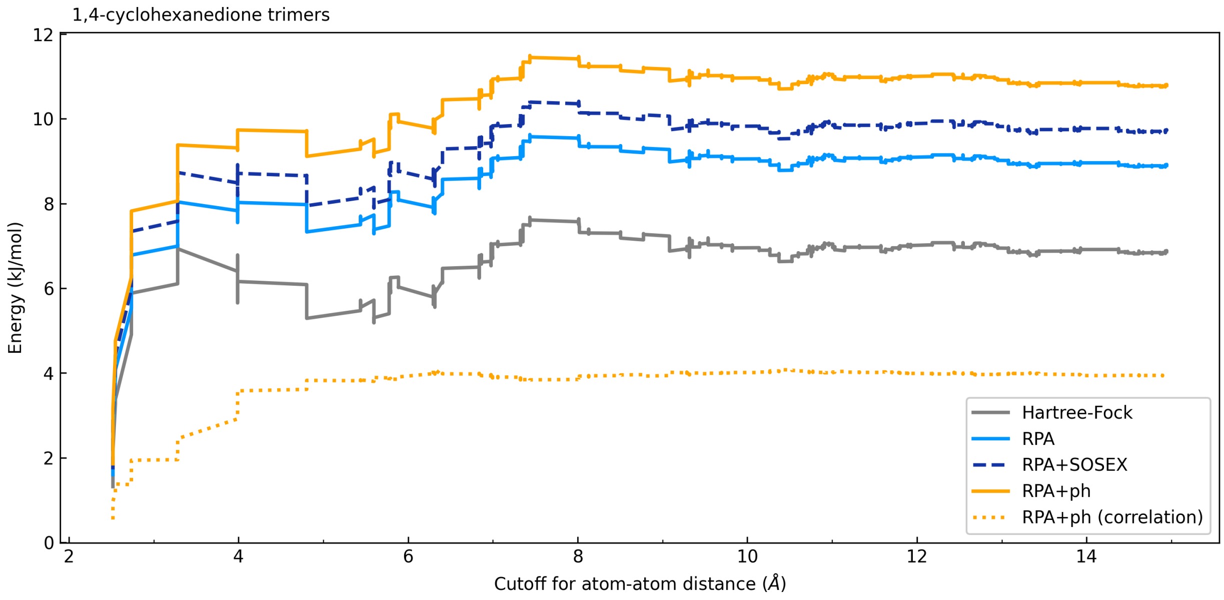

5.2 Acetic acid

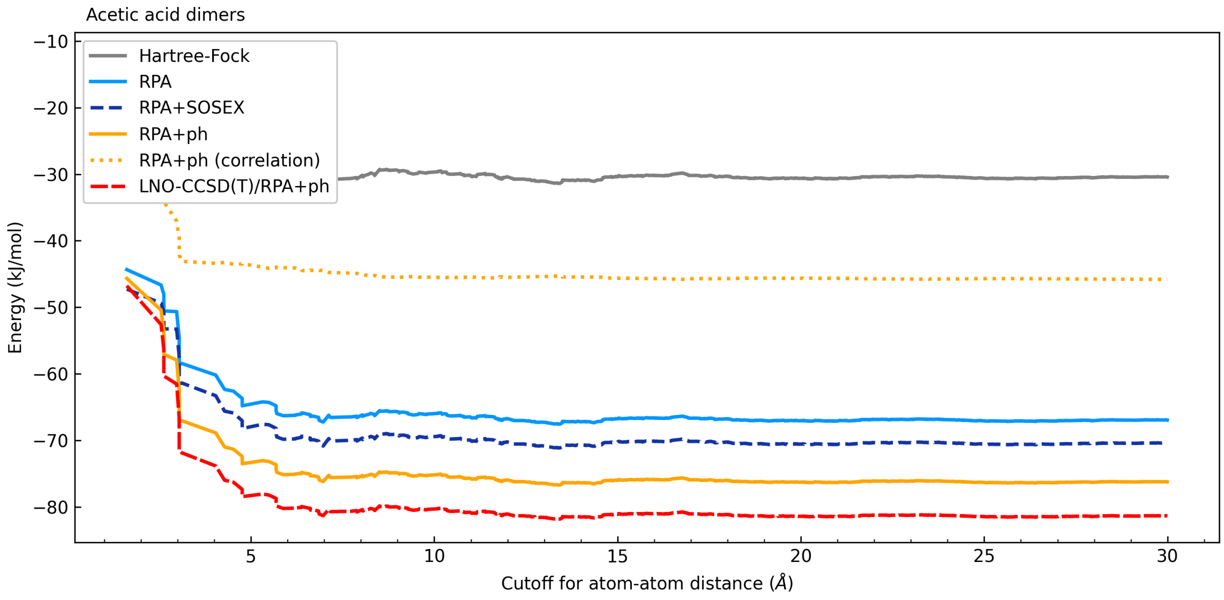

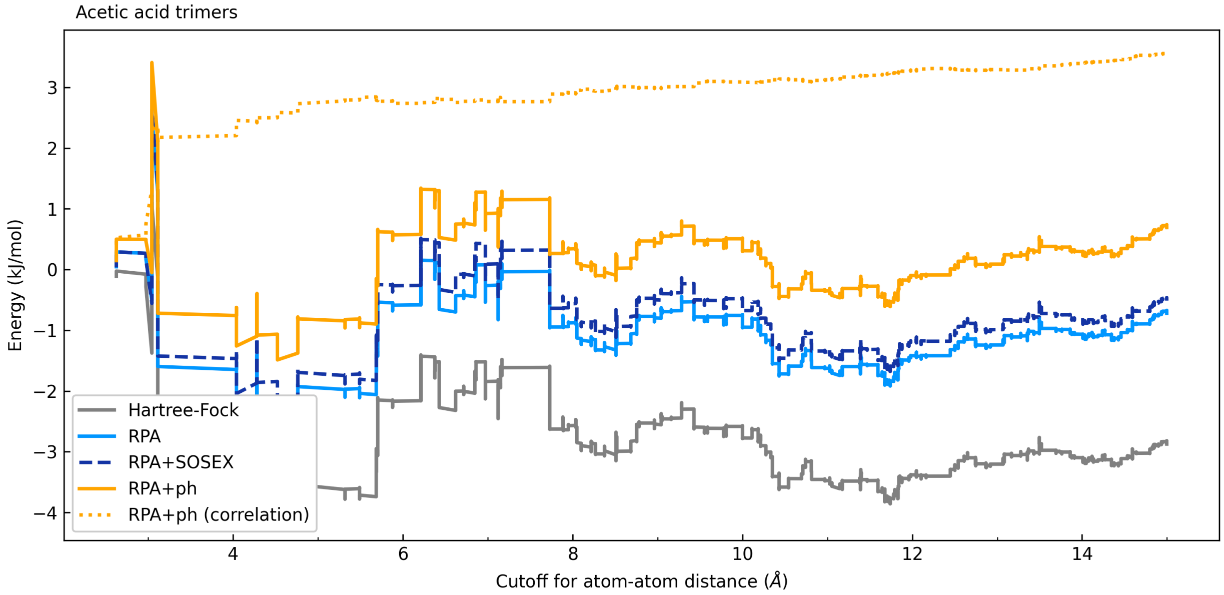

5.3 Adamantane

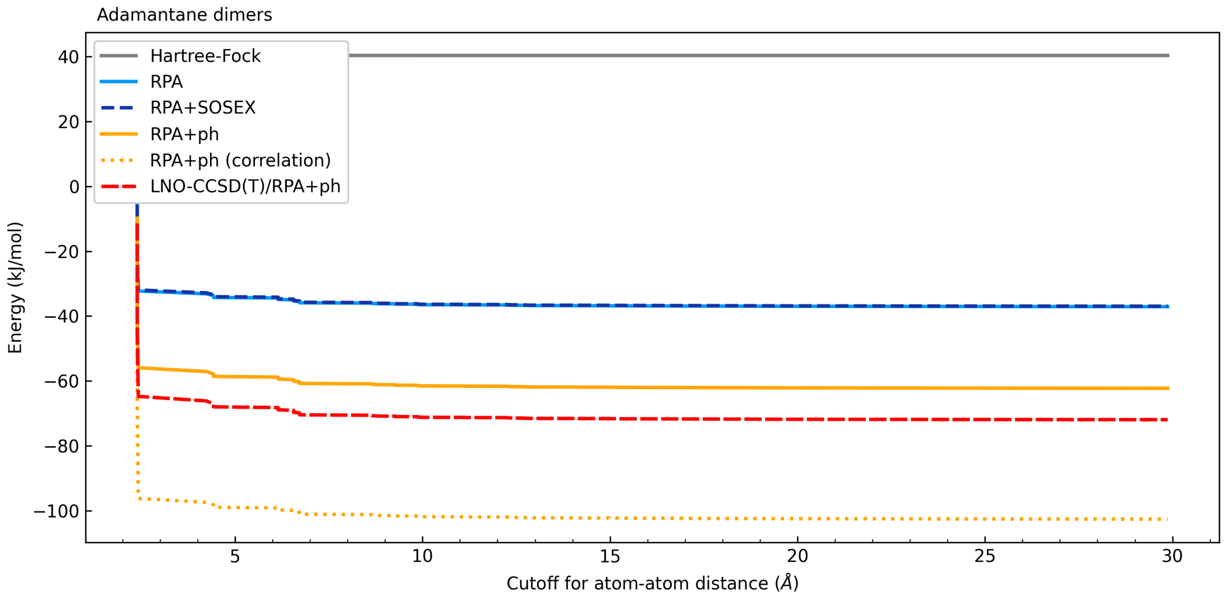

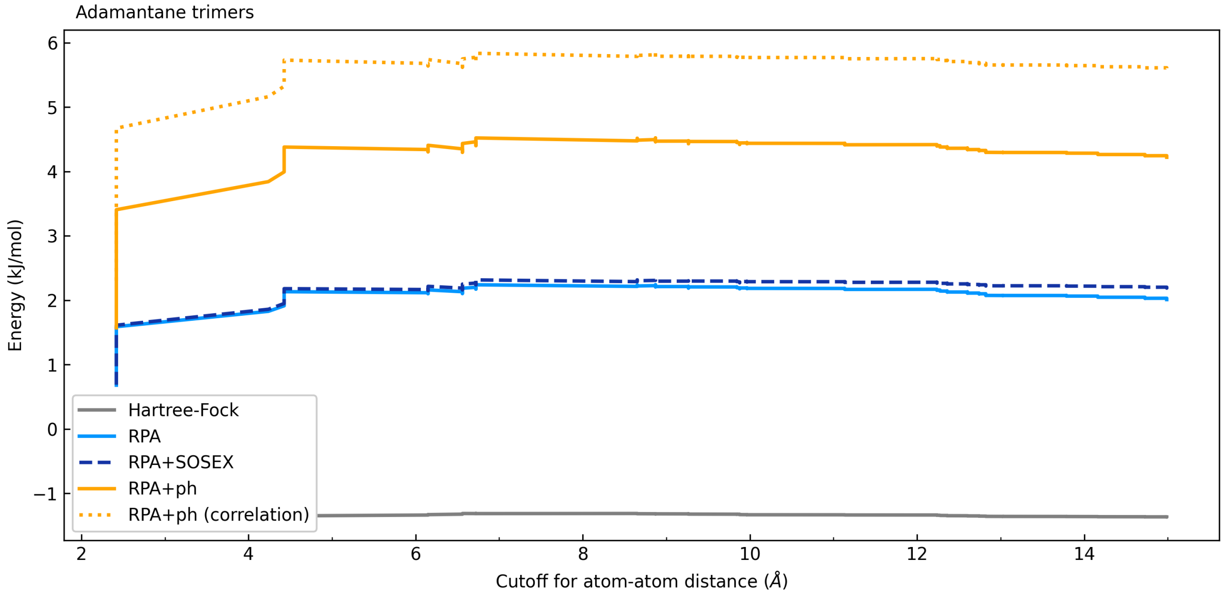

5.4 Ammonia

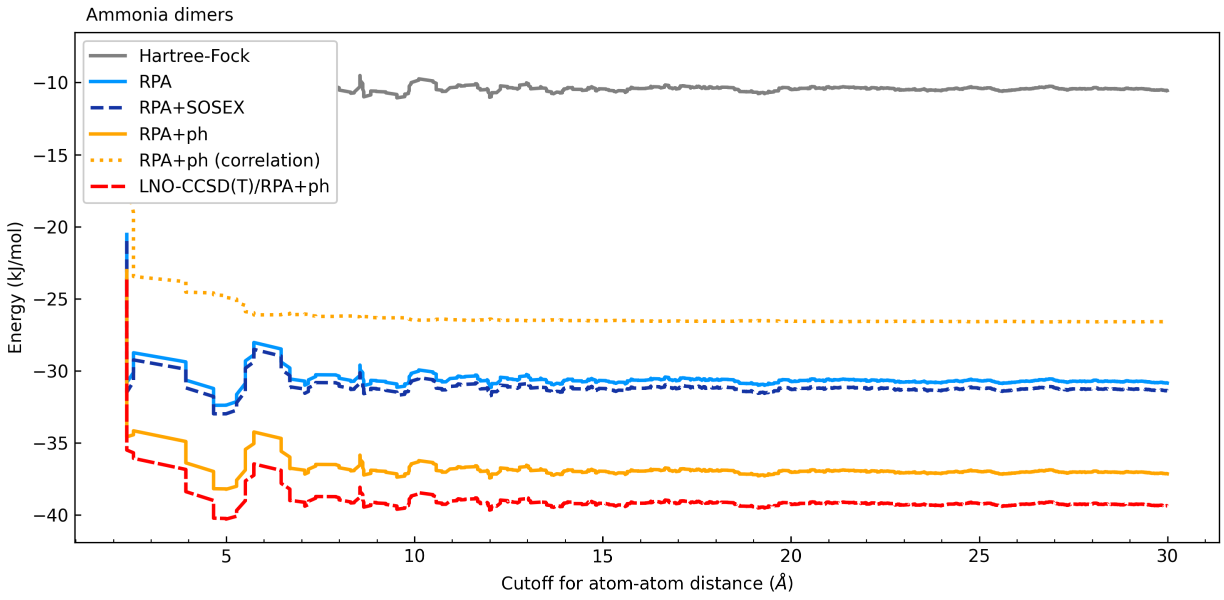
Ammonia dimers
Hartree-Fock
RPA
RPA+SOSEX
RPA+ph
RPA+ph (correlation)
LNO-CCSD(T)/RPA+ph
Energy (kJ/mol)
Cutoff for atom-atom distance (Å)

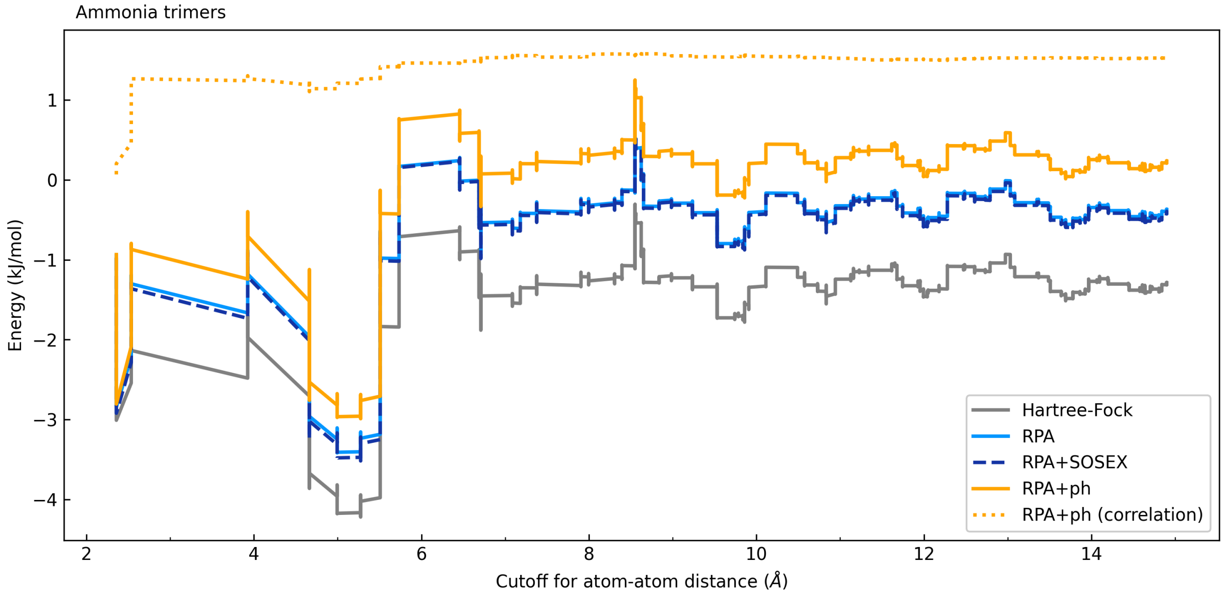
Ammonia trimers
Hartree-Fock
RPA
RPA+SOSEX
RPA+ph
RPA+ph (correlation)
Energy (kJ/mol)
Cutoff for atom-atom distance (Å)

5.5 Anthracene

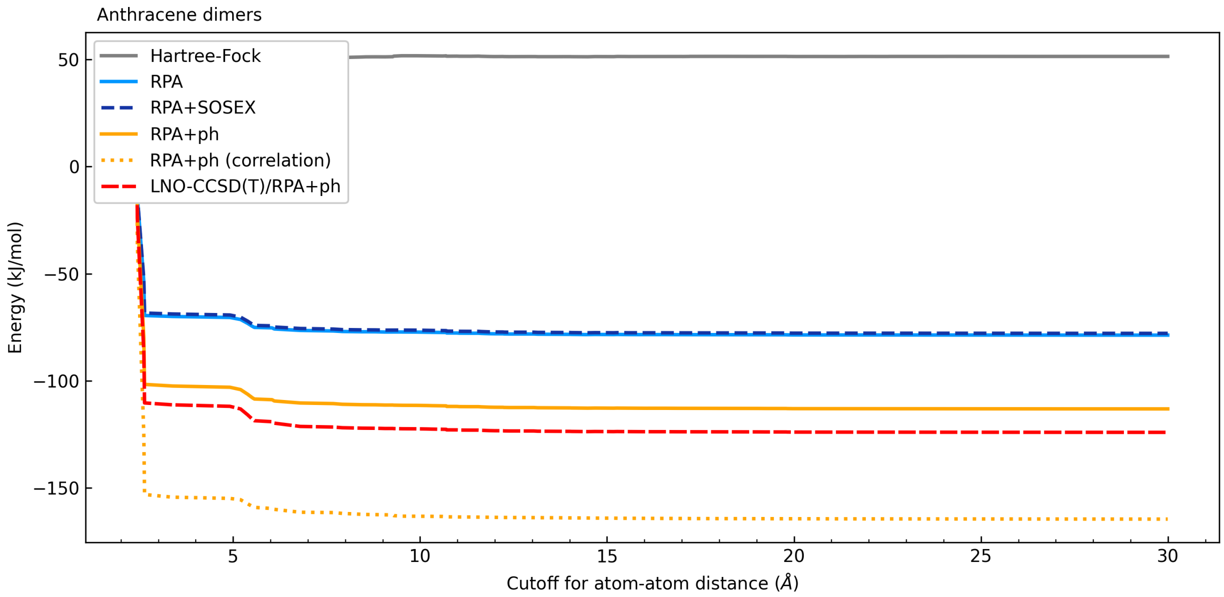

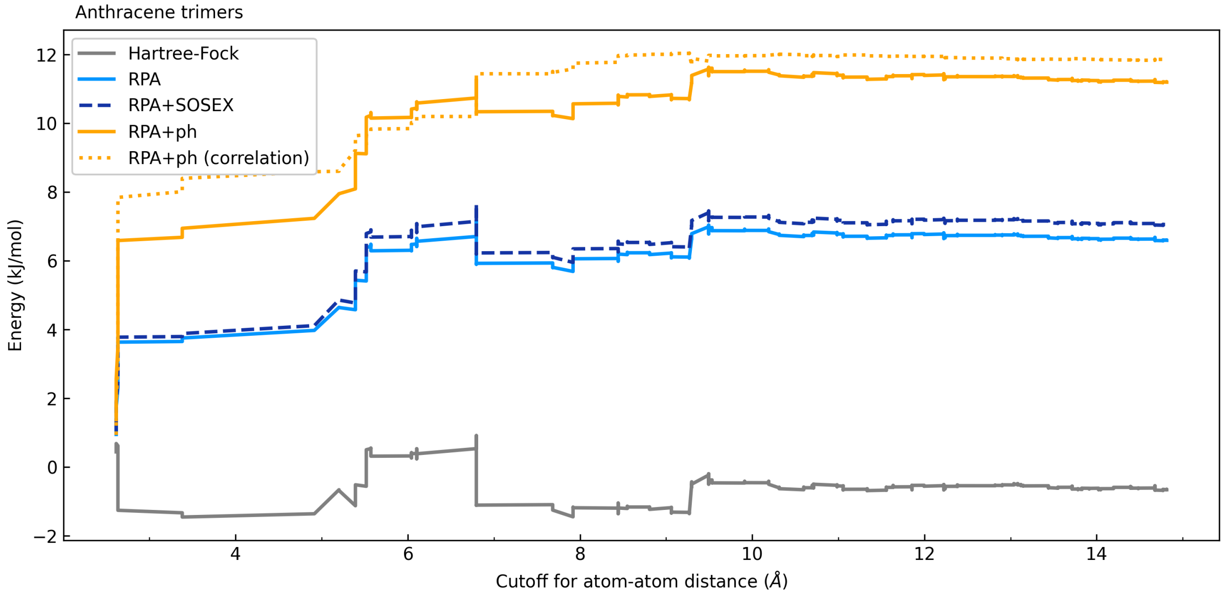

5.6 Benzene

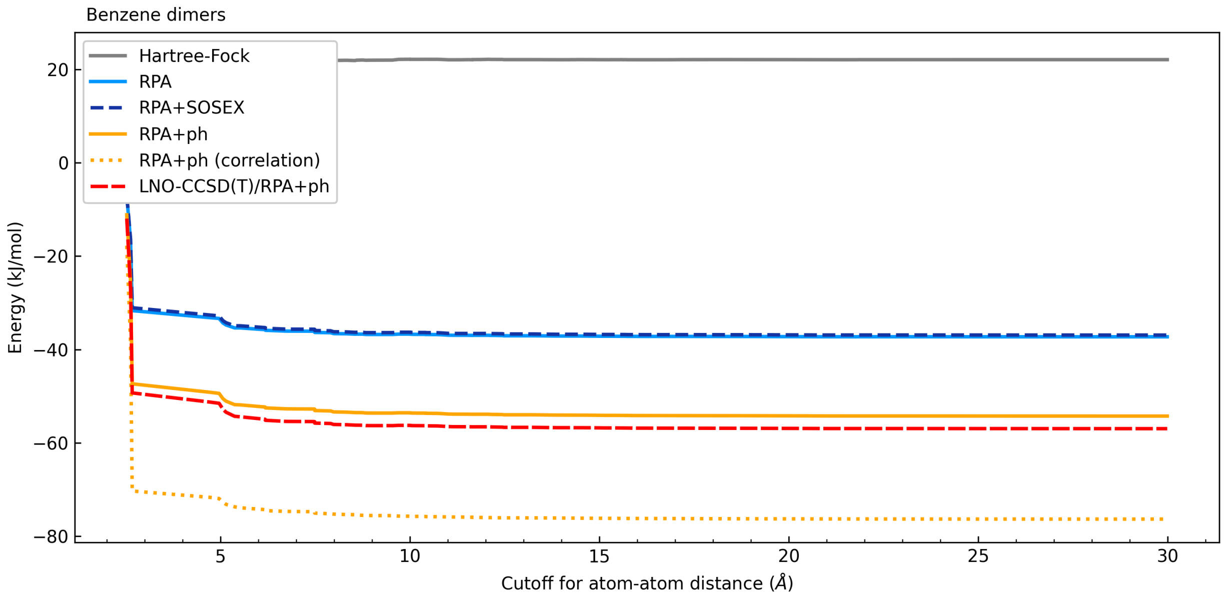

Benzene dimers
Hartree-Fock
RPA
RPA+SOSEX
RPA+ph
RPA+ph (correlation)
LNO-CCSD(T)/RPA+ph
Energy (kJ/mol)
20
0
−20
−40
−60
−80
5
10
15
20
25
30
Cutoff for atom-atom distance (Å)

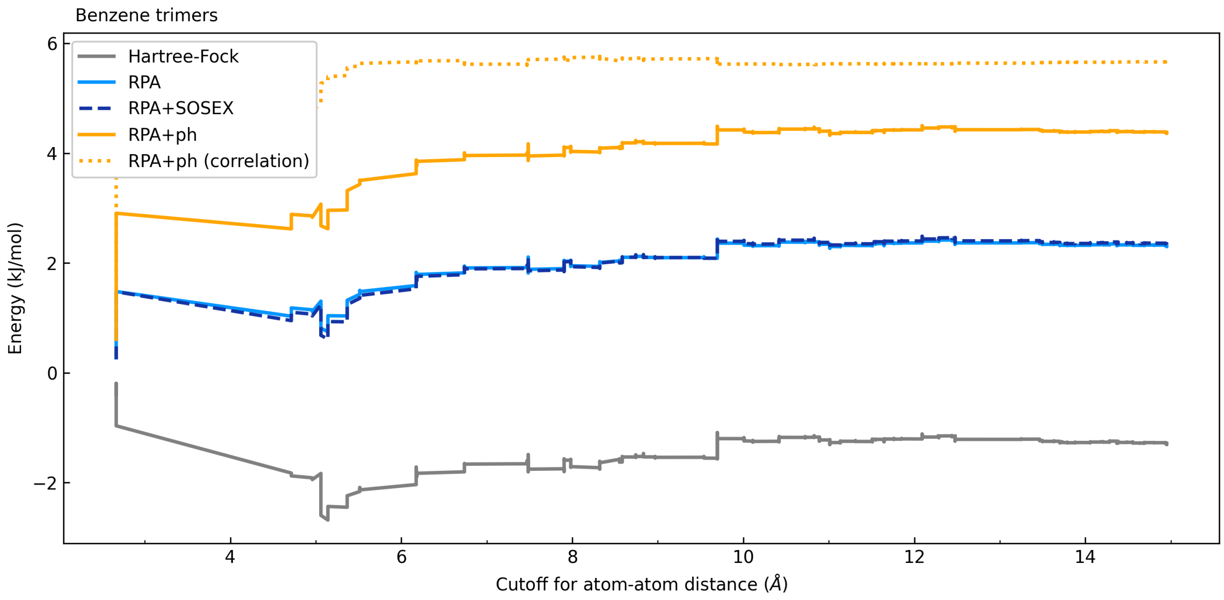

Benzene trimers
Hartree-Fock
RPA
RPA+SOSEX
RPA+ph
RPA+ph (correlation)
Energy (kJ/mol)
6
4
2
0
−2
4
6
8
10
12
14
Cutoff for atom-atom distance (Å)

5.7 CO2

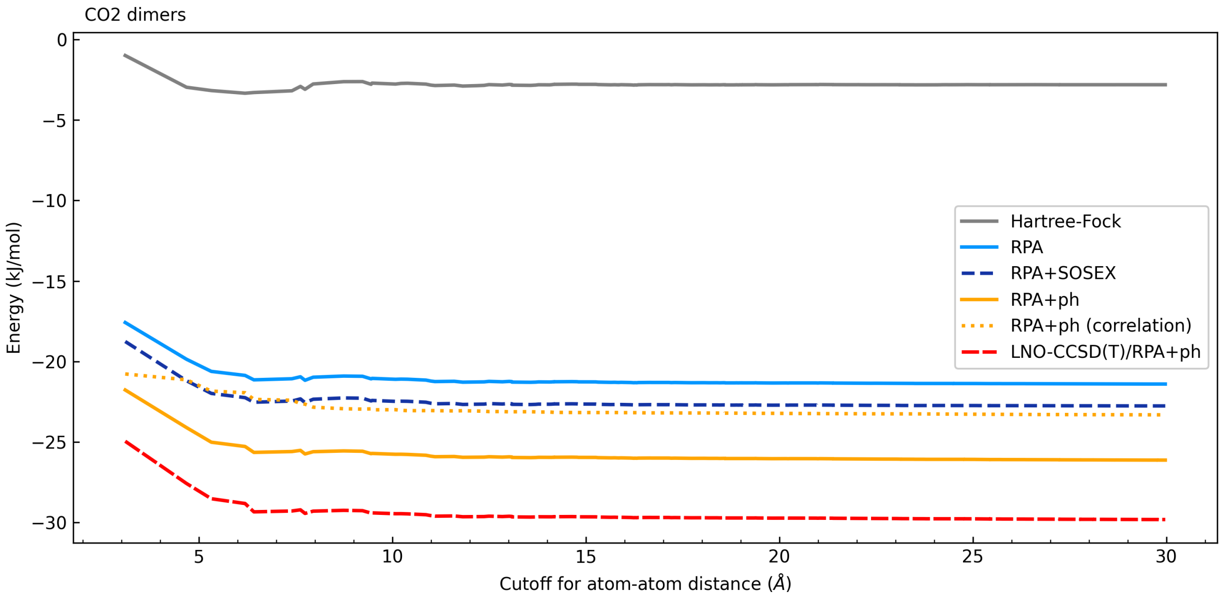

CO2 dimers
Energy (kJ/mol)
Cutoff for atom-atom distance (Å)
0
−5
−10
−15
−20
−25
−30
5
10
15
20
25
30
Hartree-Fock
RPA
RPA+SOSEX
RPA+ph
RPA+ph (correlation)
LNO-CCSD(T)/RPA+ph

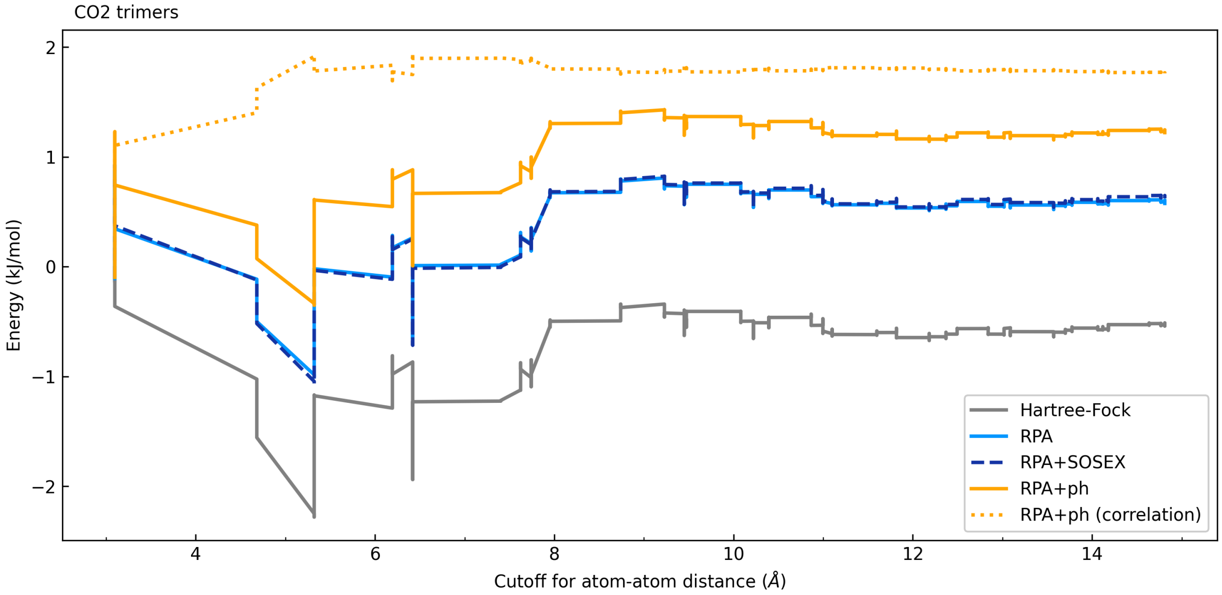

CO2 trimers
Energy (kJ/mol)
Cutoff for atom-atom distance (Å)
2
1
0
−1
−2
4
6
8
10
12
14
Hartree-Fock
RPA
RPA+SOSEX
RPA+ph
RPA+ph (correlation)

5.8 Cyanamide

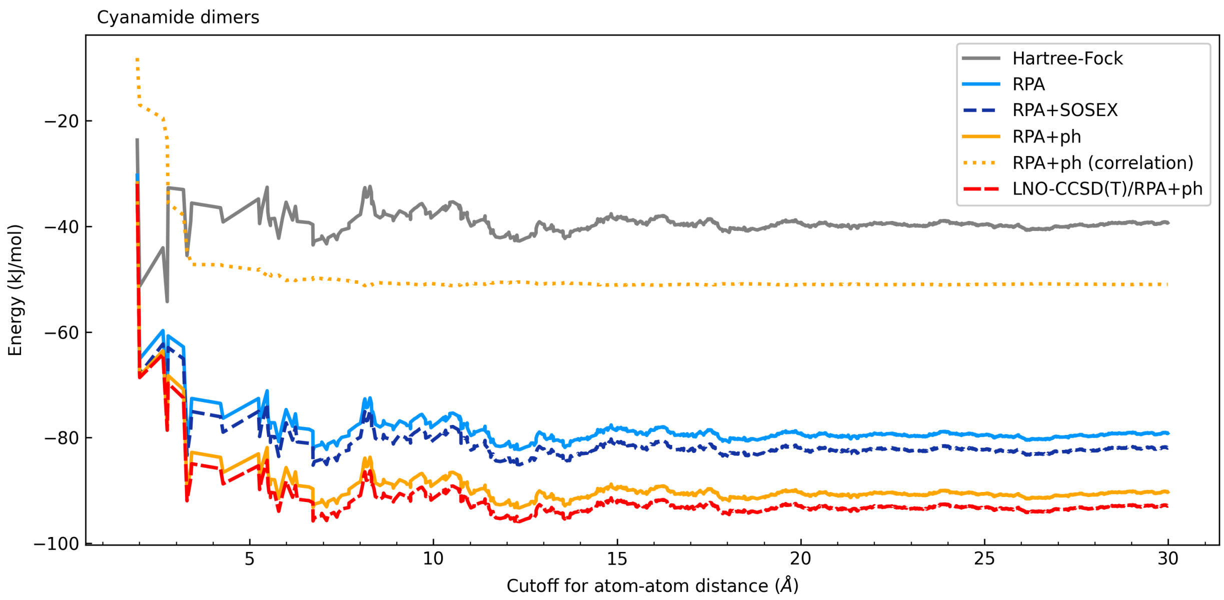

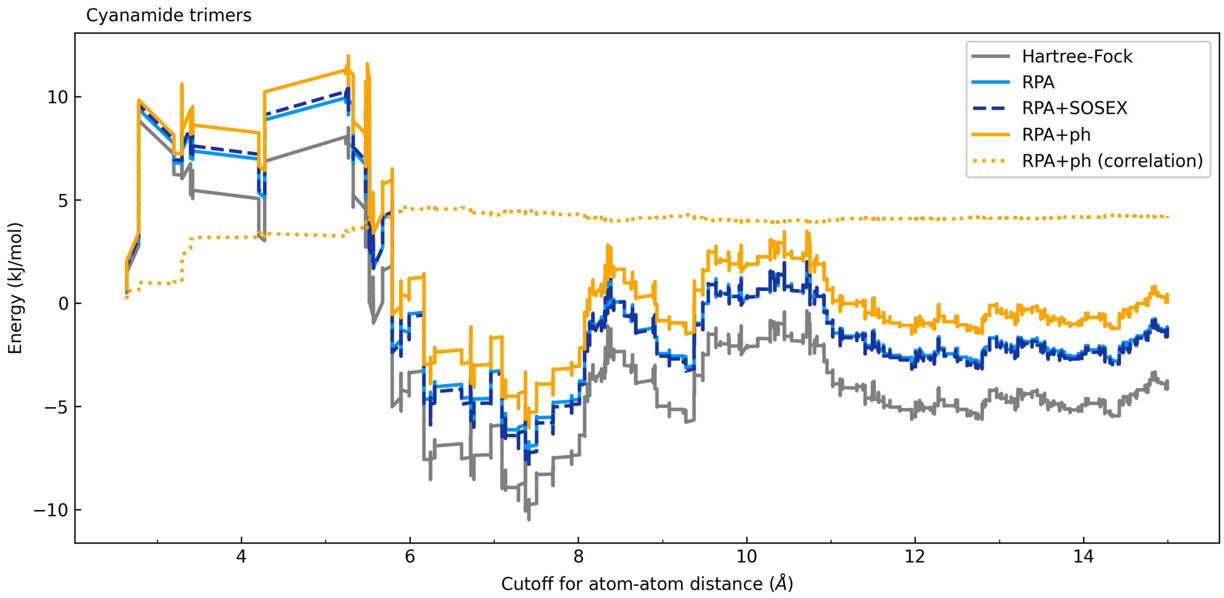

5.9 Cytosine

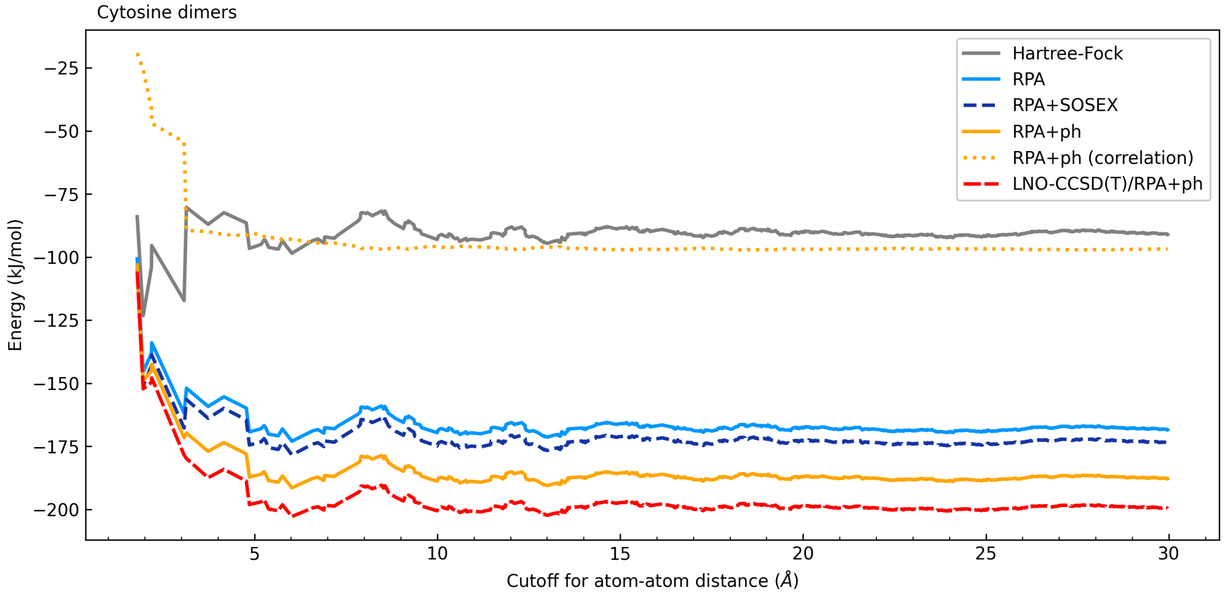

Cytosine dimers
Hartree-Fock
RPA
RPA+SOSEX
RPA+ph
RPA+ph (correlation)
LNO-CCSD(T)/RPA+ph
Energy (kJ/mol)
−25
−50
−75
−100
−125
−150
−175
−200
5
10
15
20
25
30
Cutoff for atom-atom distance (Å)

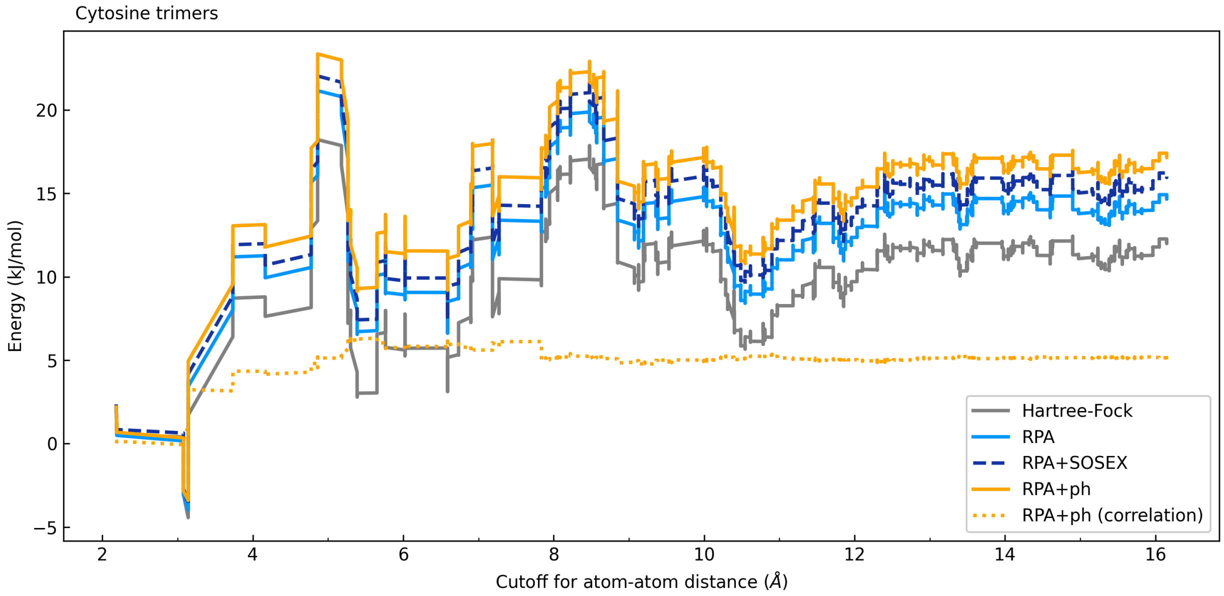

Cytosine trimers
Hartree-Fock
RPA
RPA+SOSEX
RPA+ph
RPA+ph (correlation)
Energy (kJ/mol)
20
15
10
5
0
−5
2
4
6
8
10
12
14
16
Cutoff for atom-atom distance (Å)

5.10 Ethyl carbamate

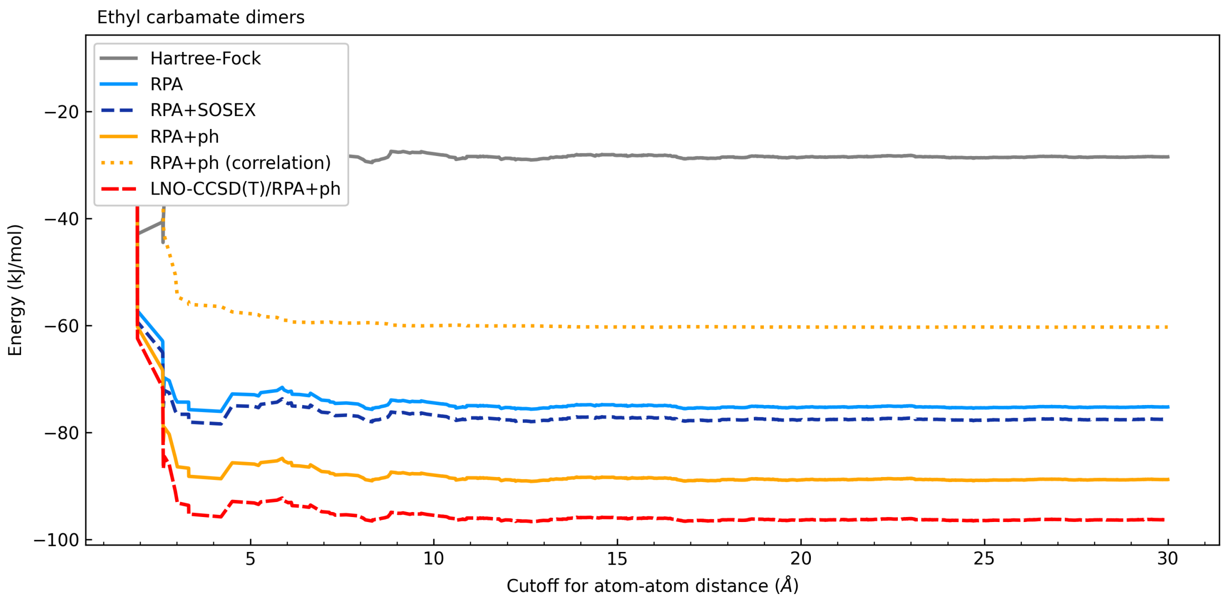

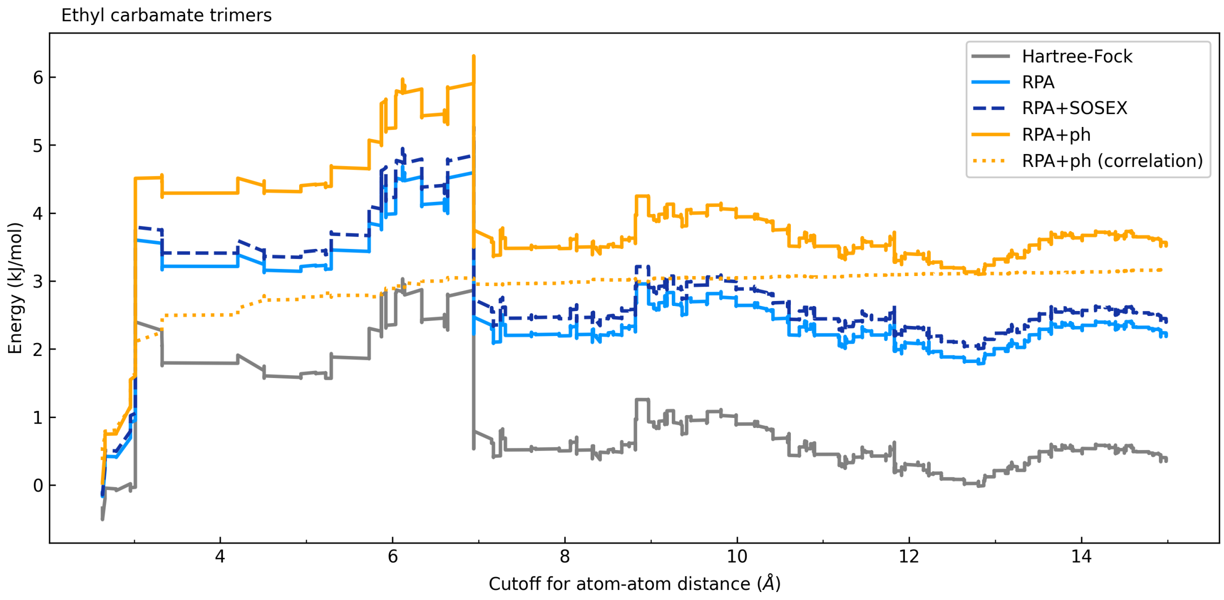

5.11 Formamide

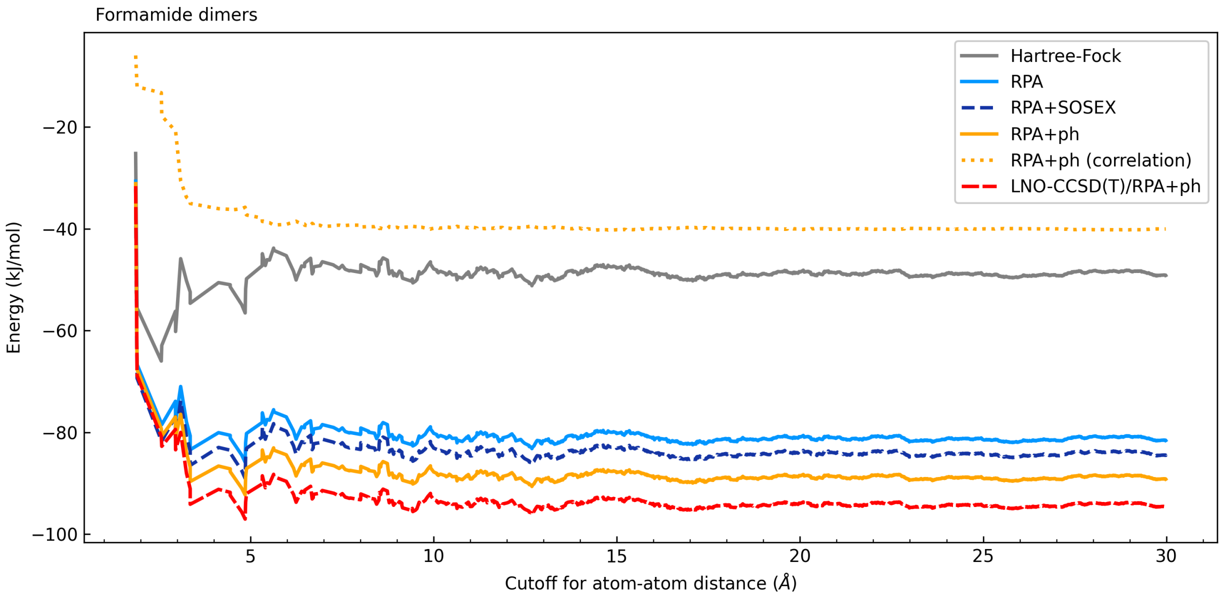

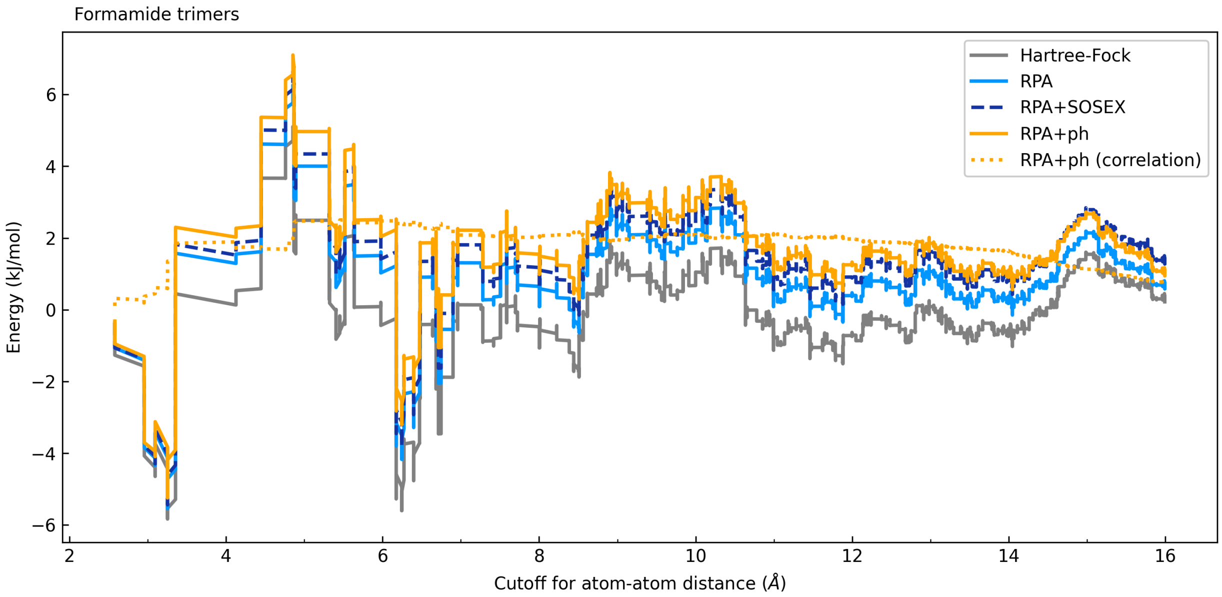

5.12 Idazole

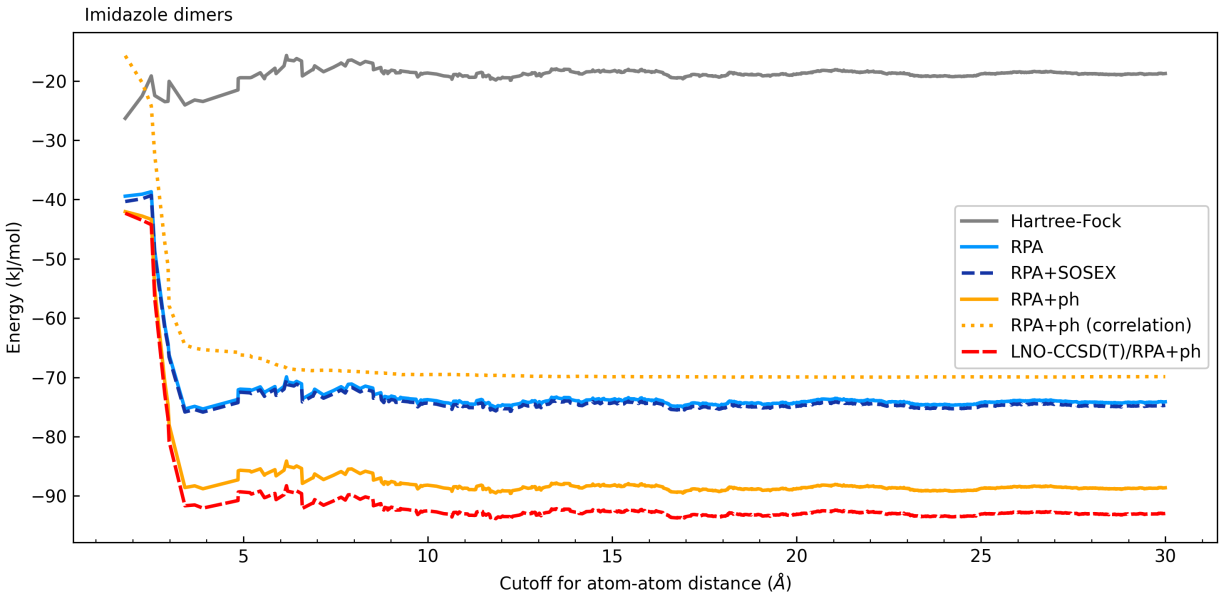

Imidazole dimers
Energy (kJ/mol)
−20
−30
−40
−50
−60
−70
−80
−90
5
10
15
20
25
30
Cutoff for atom-atom distance (Å)
Hartree-Fock
RPA
RPA+SOSEX
RPA+ph
RPA+ph (correlation)
LNO-CCSD(T)/RPA+ph

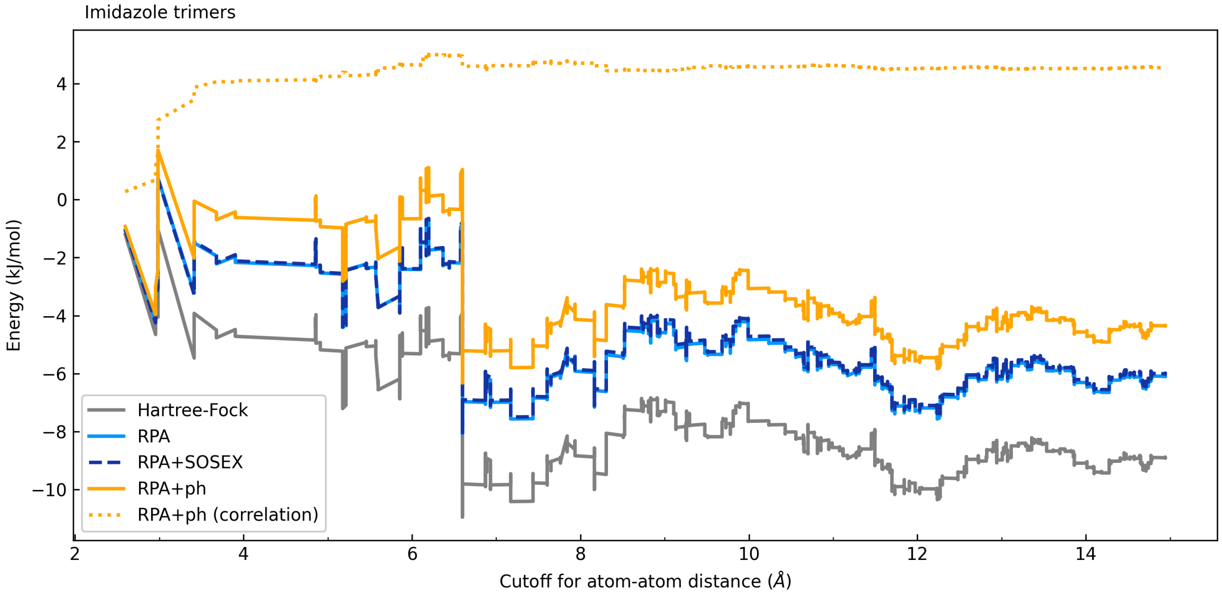

Imidazole trimers
Energy (kJ/mol)
4
2
0
−2
−4
−6
−8
−10
2
4
6
8
10
12
14
Cutoff for atom-atom distance (Å)
Hartree-Fock
RPA
RPA+SOSEX
RPA+ph
RPA+ph (correlation)

5.13 Naphthalene

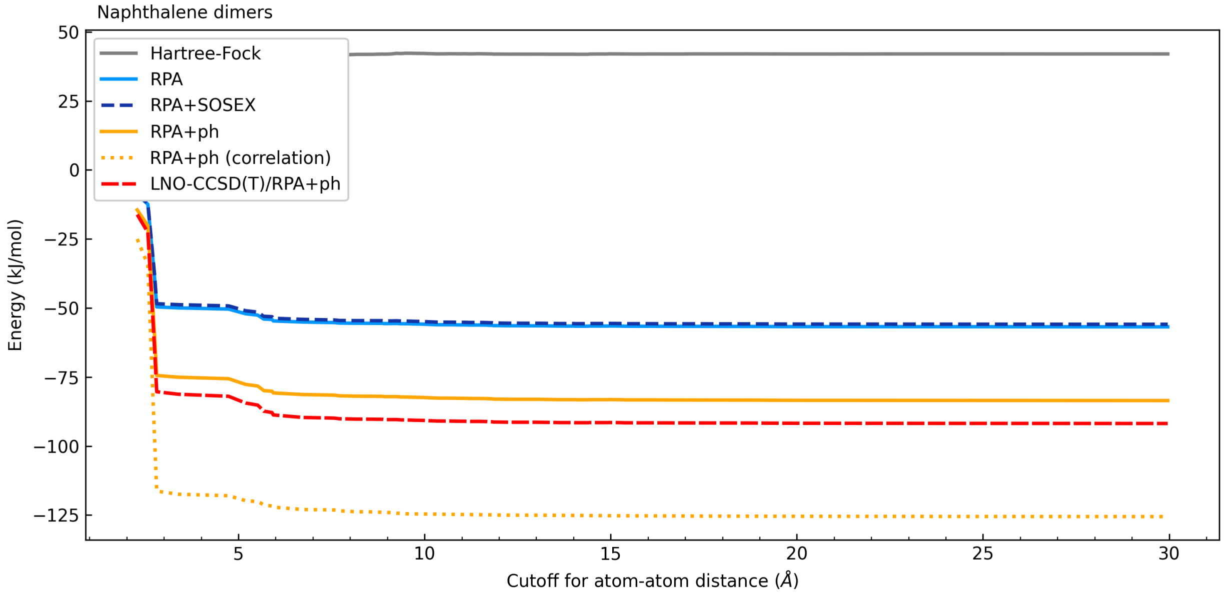

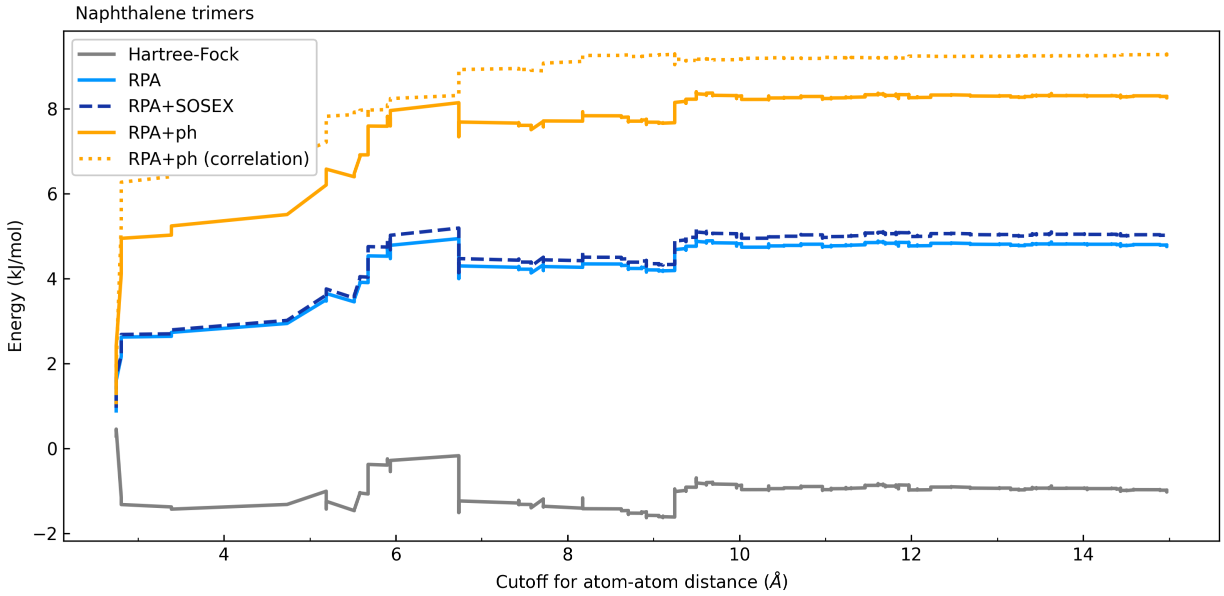

5.14 Oxalic acid α

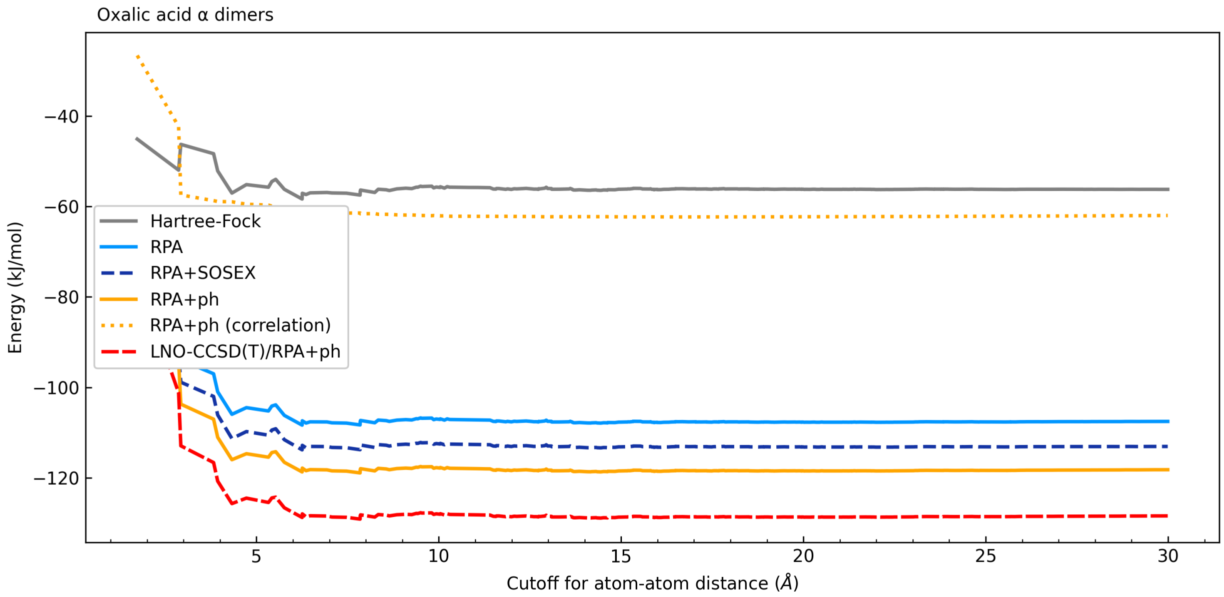

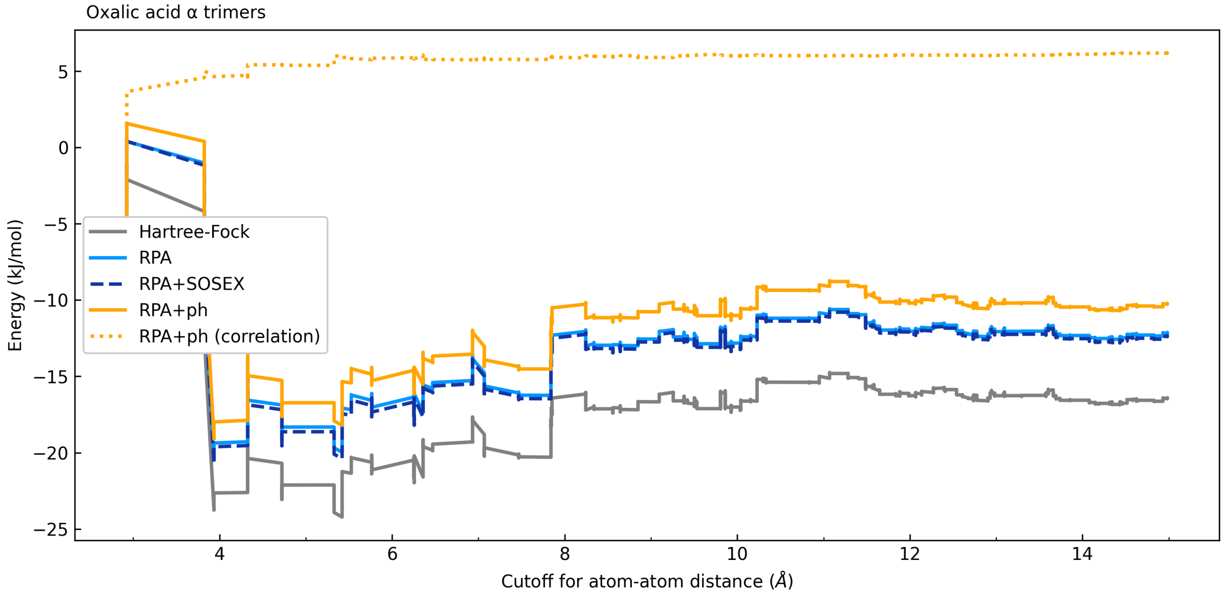

5.15 Oxalic acid β

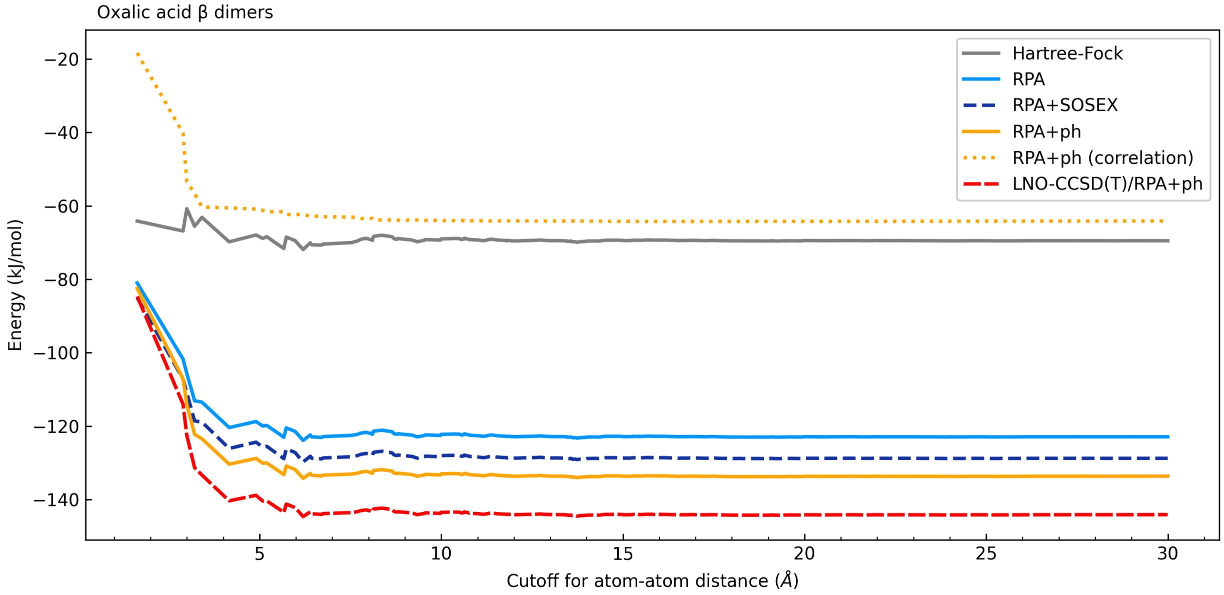

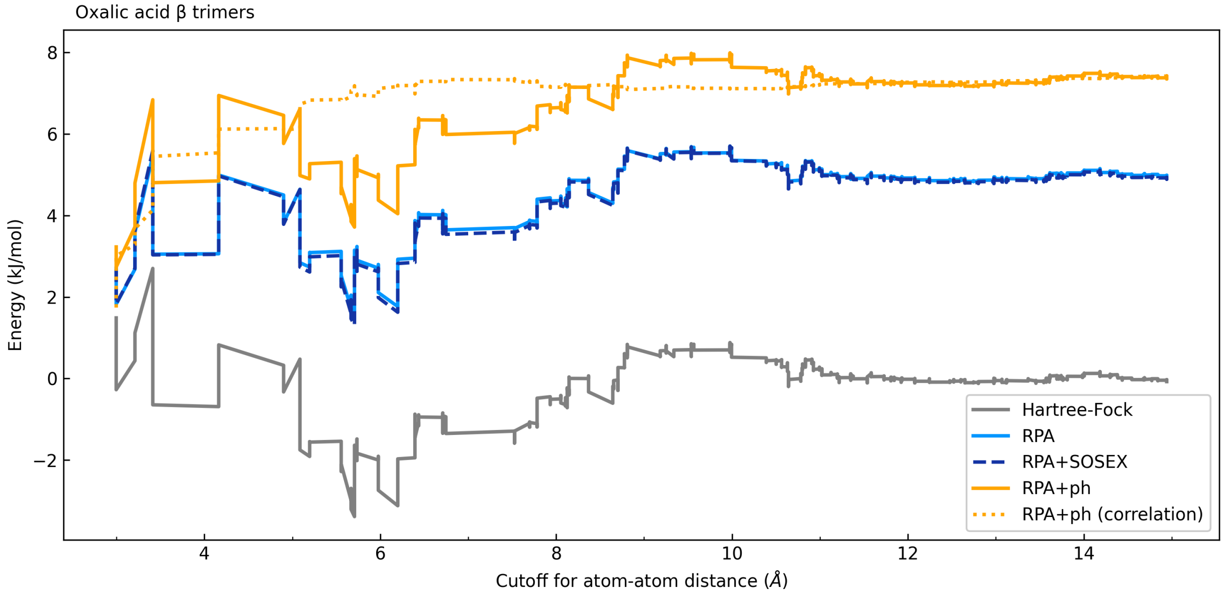

5.16 Pyrazine

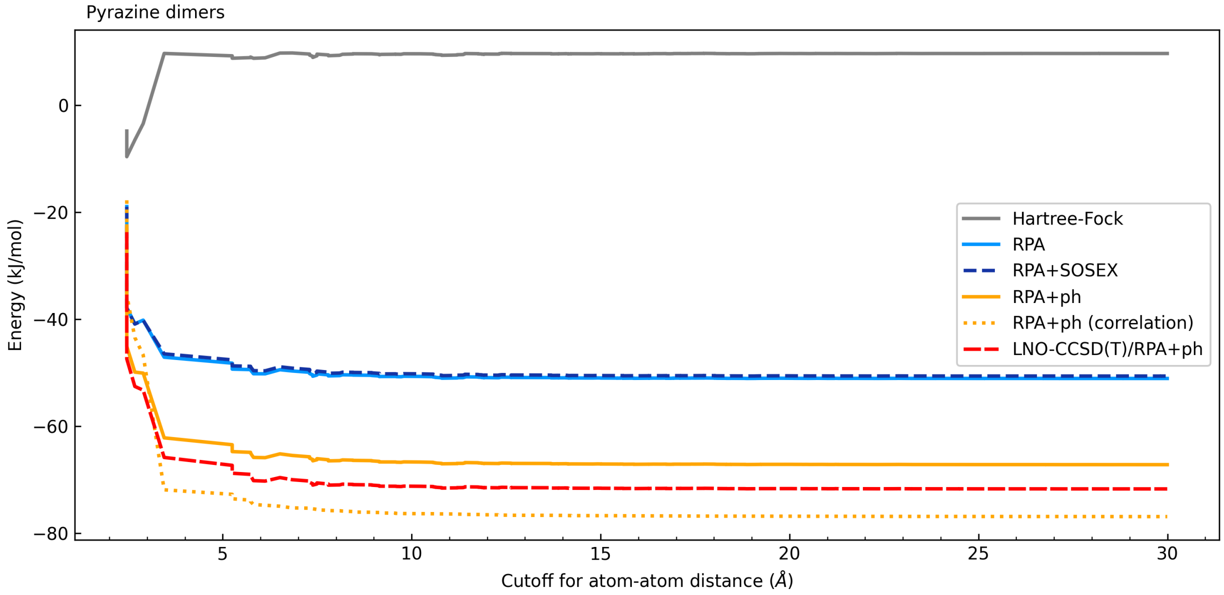

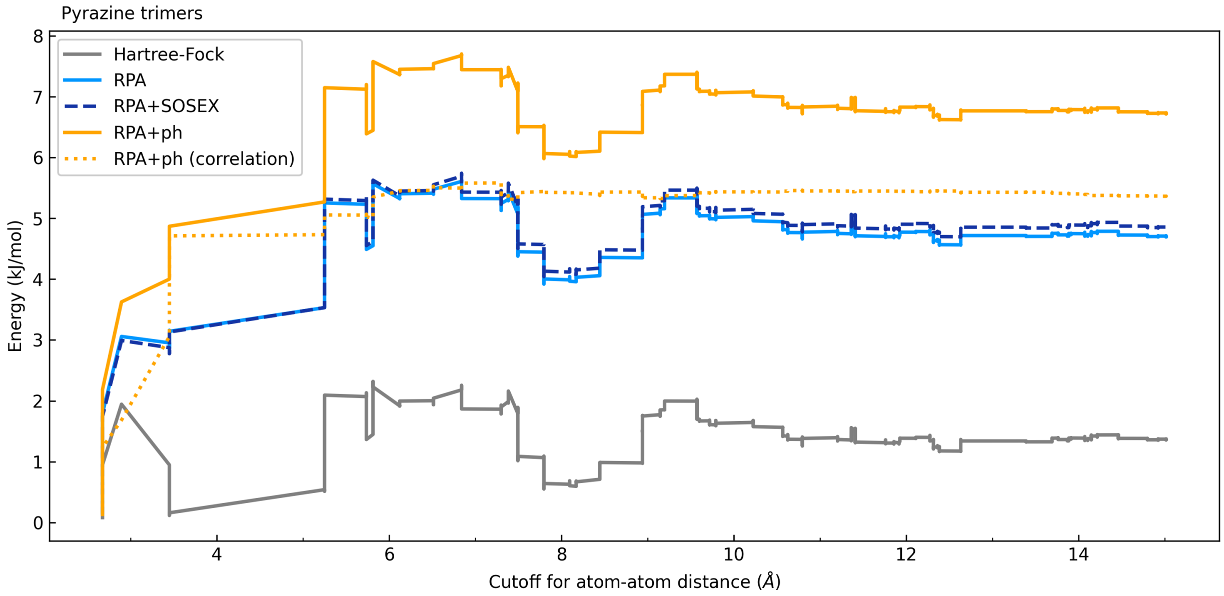

5.17 Pyrazole

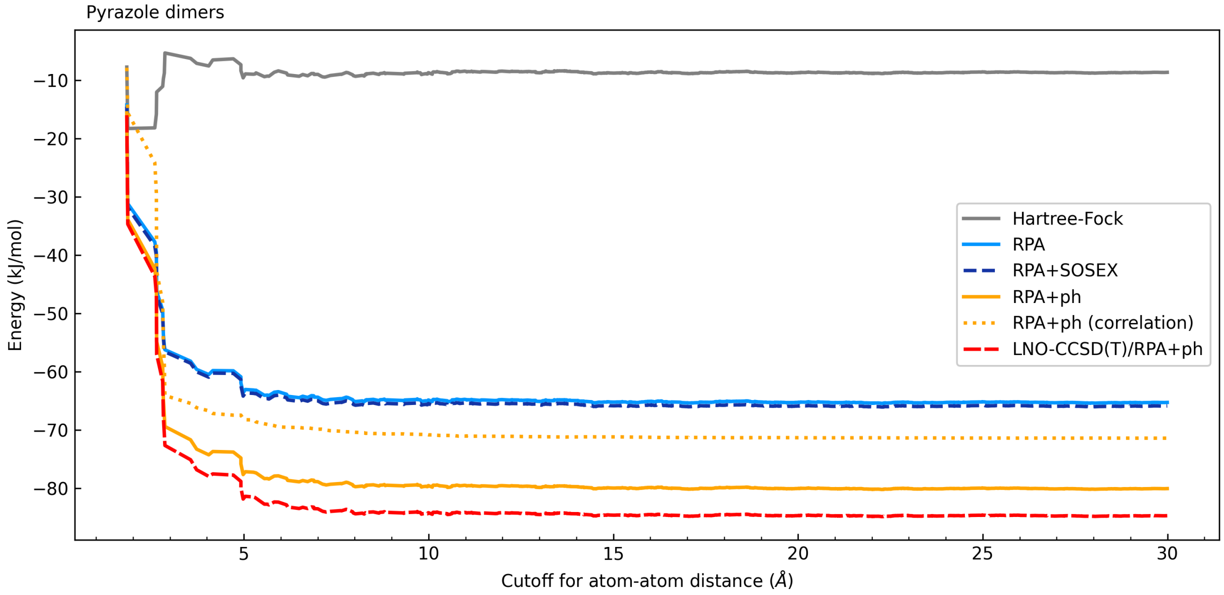

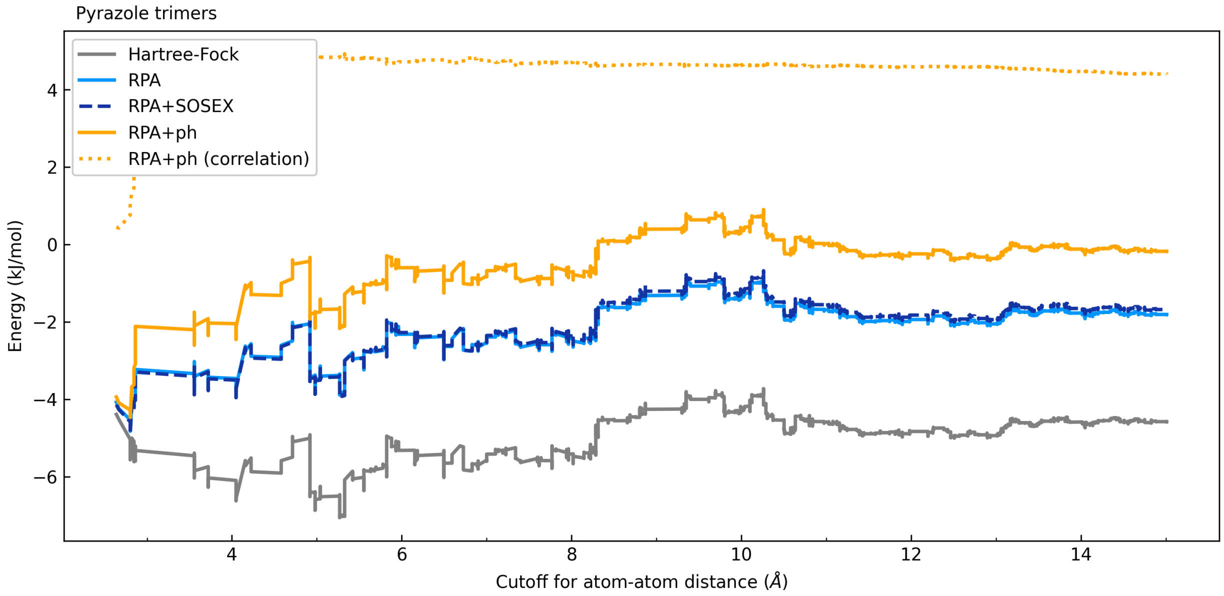

5.18 Triazine

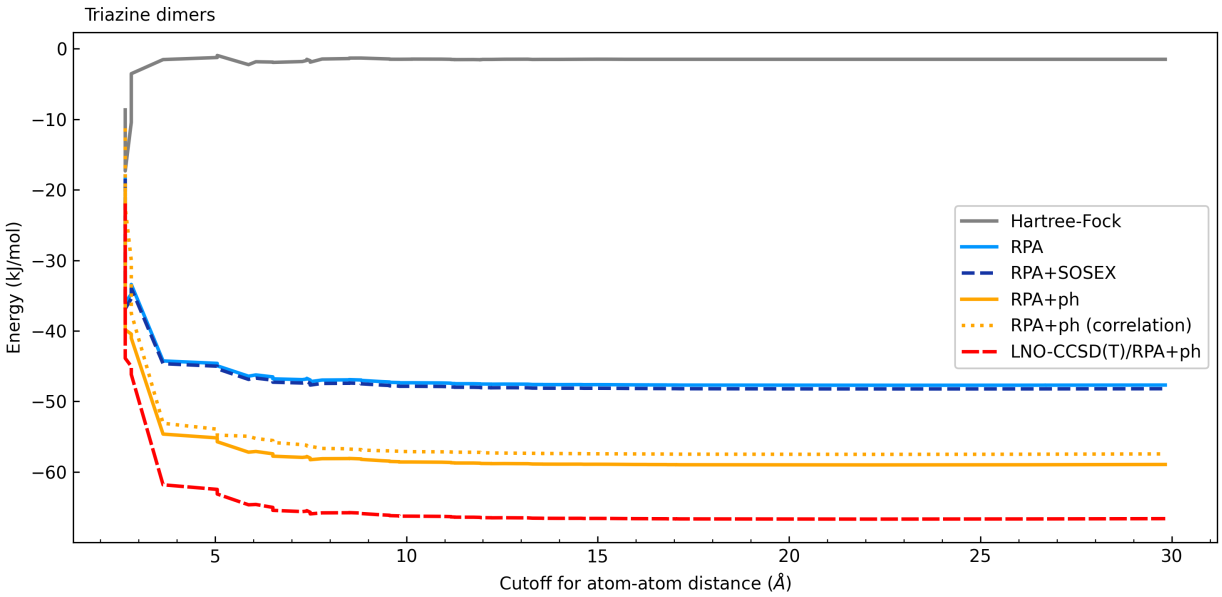

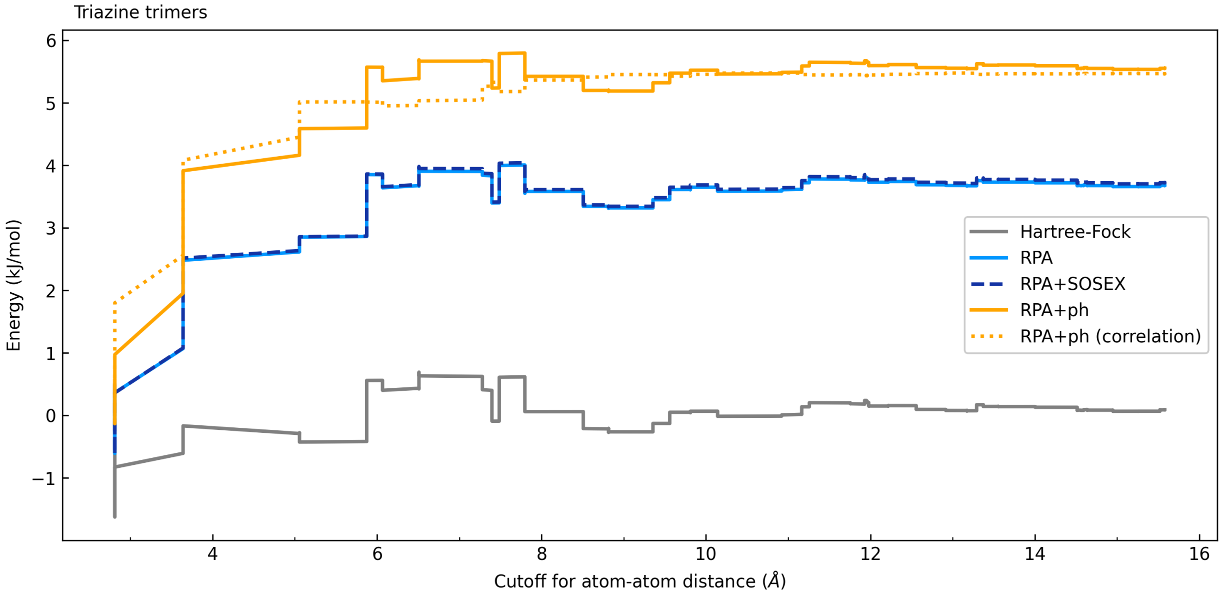

5.19 Trioxane

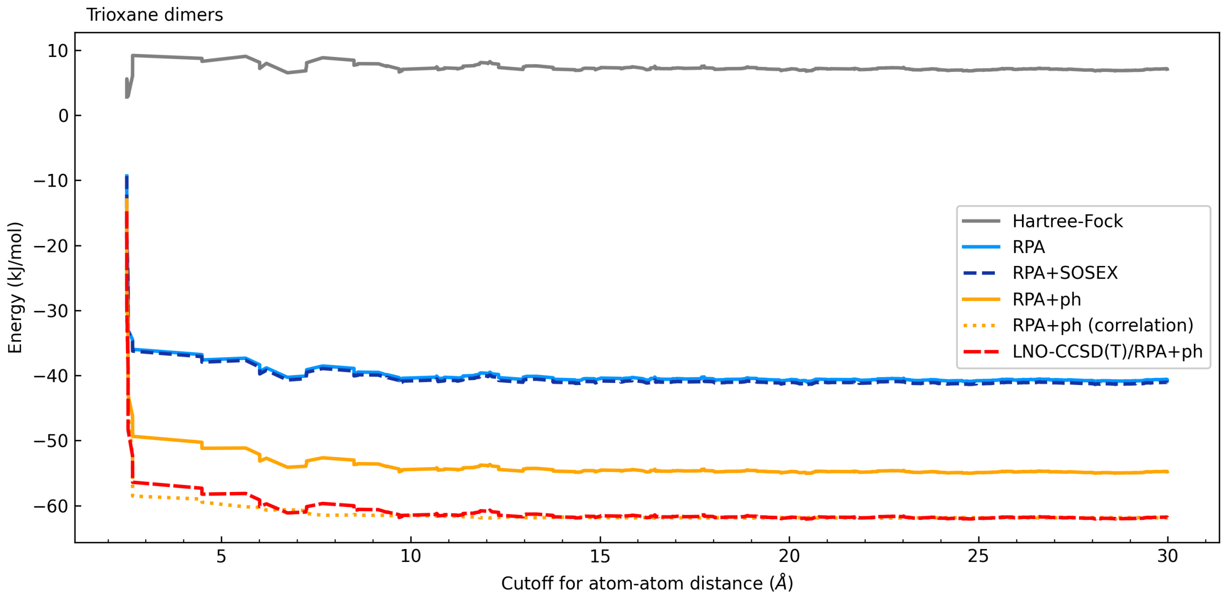

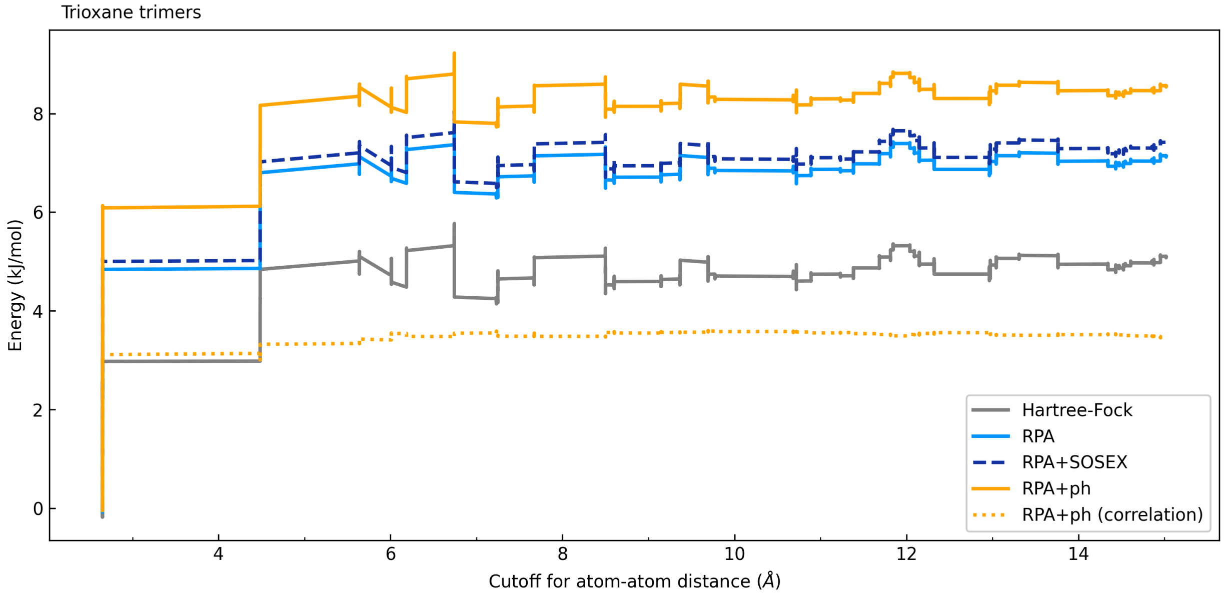

5.20 Uracil

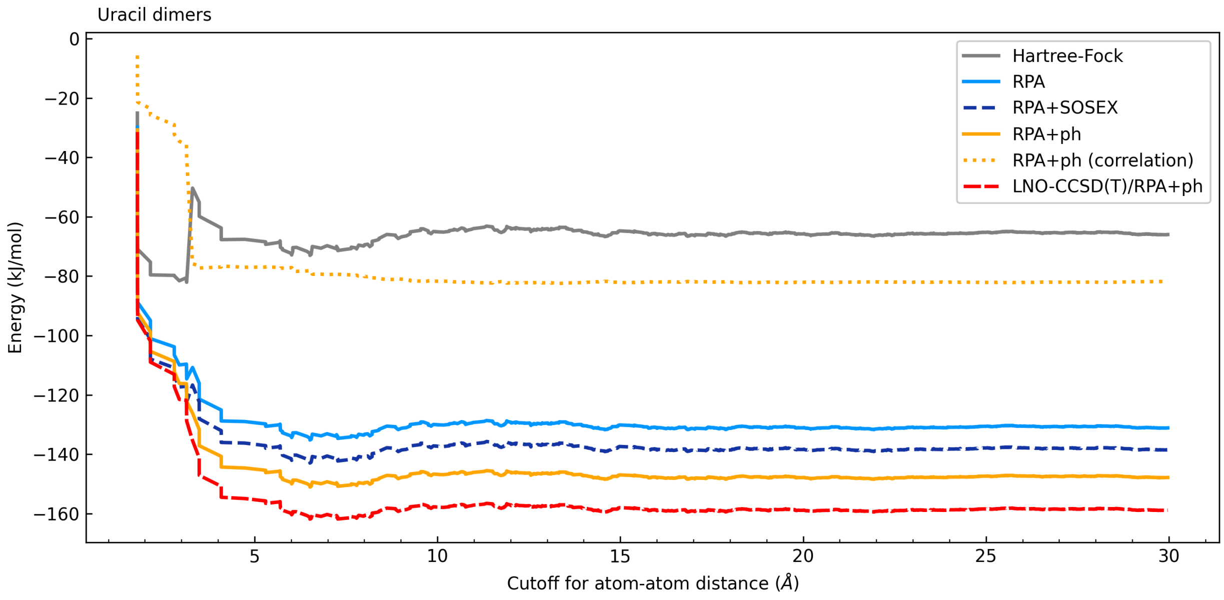
Uracil dimers
Energy (kJ/mol)
0
−20
−40
−60
−80
−100
−120
−140
−160
5
10
15
20
25
30
Cutoff for atom-atom distance (Å)
Hartree-Fock
RPA
RPA+SOSEX
RPA+ph
RPA+ph (correlation)
LNO-CCSD(T)/RPA+ph

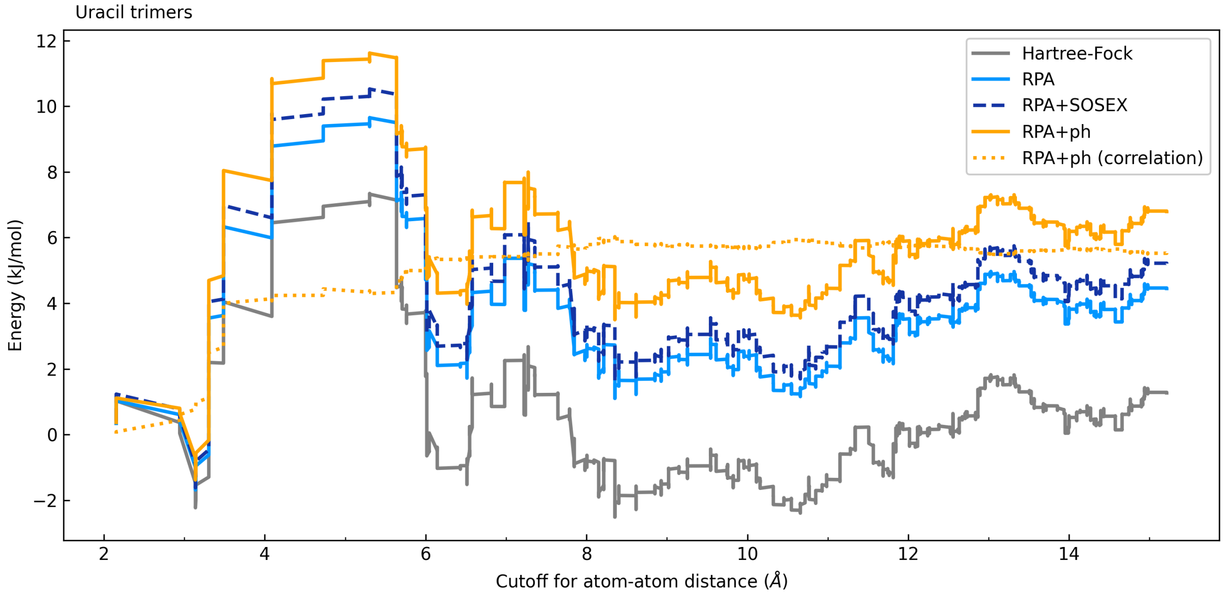
Uracil trimers
Energy (kJ/mol)
12
10
8
6
4
2
0
−2
2
4
6
8
10
12
14
Cutoff for atom-atom distance (Å)
Hartree-Fock
RPA
RPA+SOSEX
RPA+ph
RPA+ph (correlation)

5.21 Urea

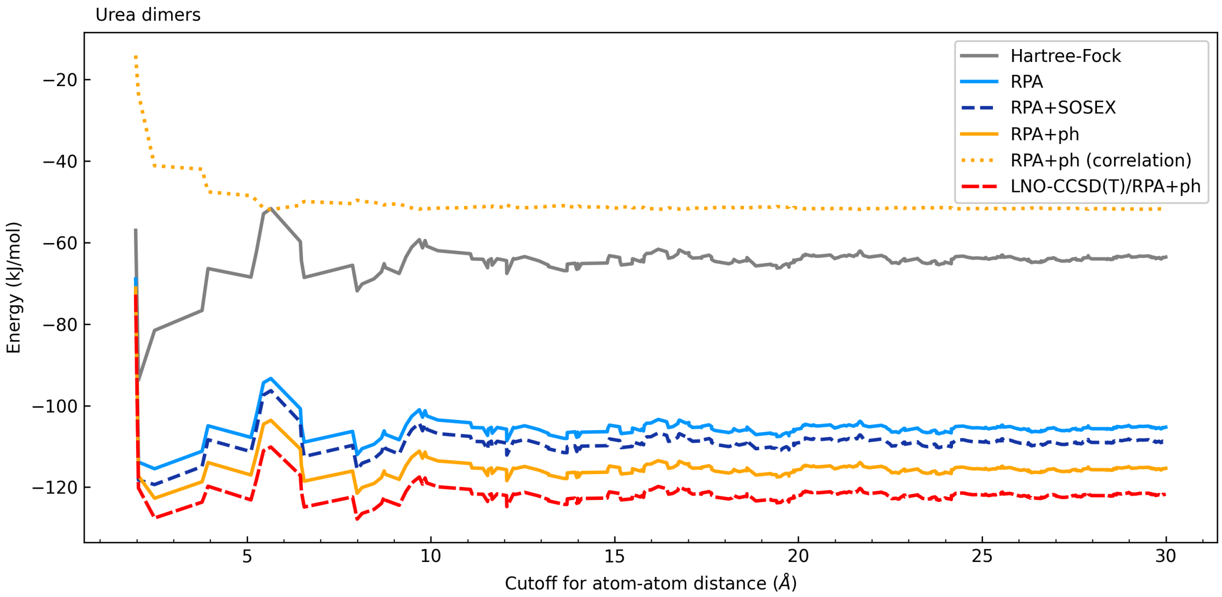

Urea dimers
Energy (kJ/mol)
−20
−40
−60
−80
−100
−120
5
10
15
20
25
30
Cutoff for atom-atom distance (Å)
Hartree-Fock
RPA
RPA+SOSEX
RPA+ph
RPA+ph (correlation)
LNO-CCSD(T)/RPA+ph

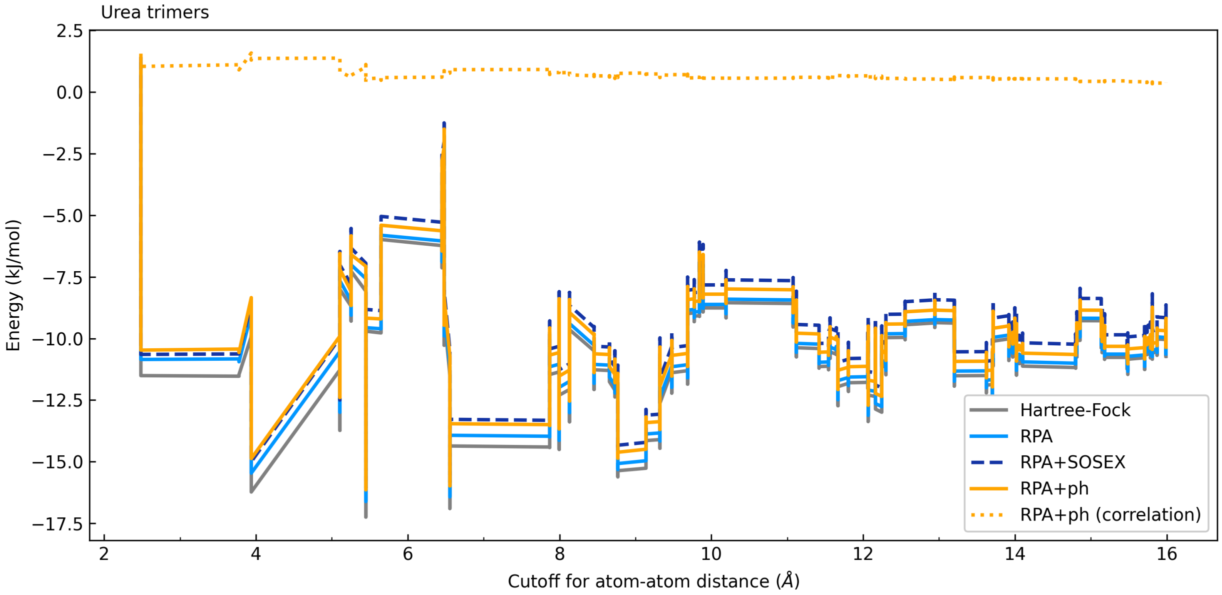

Urea trimers
Energy (kJ/mol)
2.5
0.0
−2.5
−5.0
−7.5
−10.0
−12.5
−15.0
−17.5
2
4
6
8
10
12
14
16
Cutoff for atom-atom distance (Å)
Hartree-Fock
RPA
RPA+SOSEX
RPA+ph
RPA+ph (correlation)

5.22 Hexamethylenetetramine

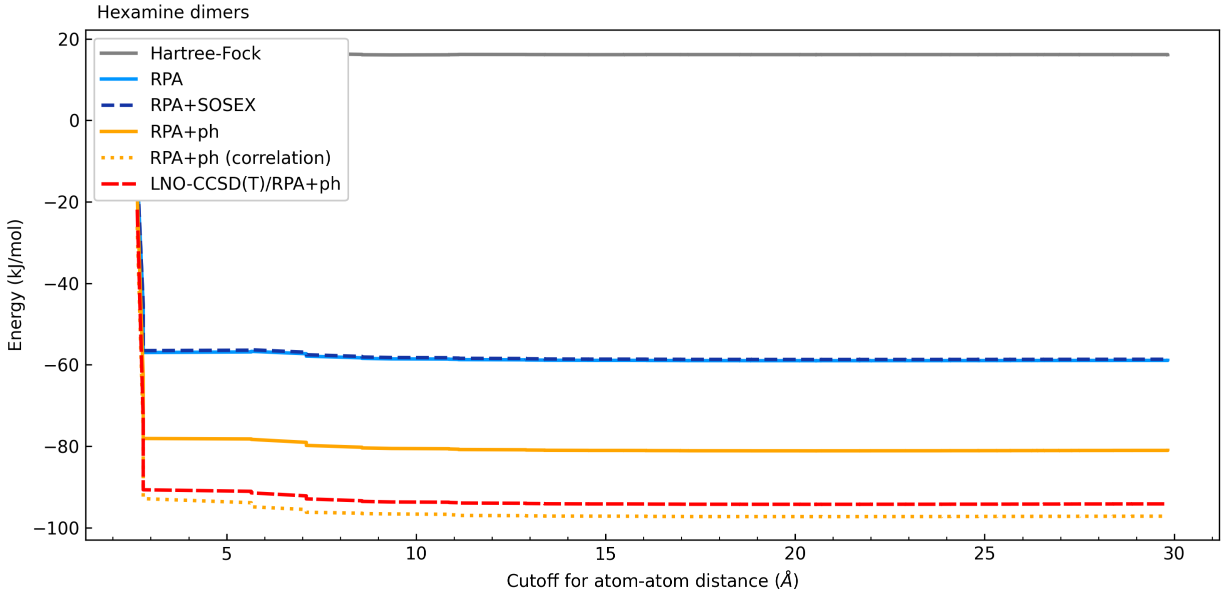

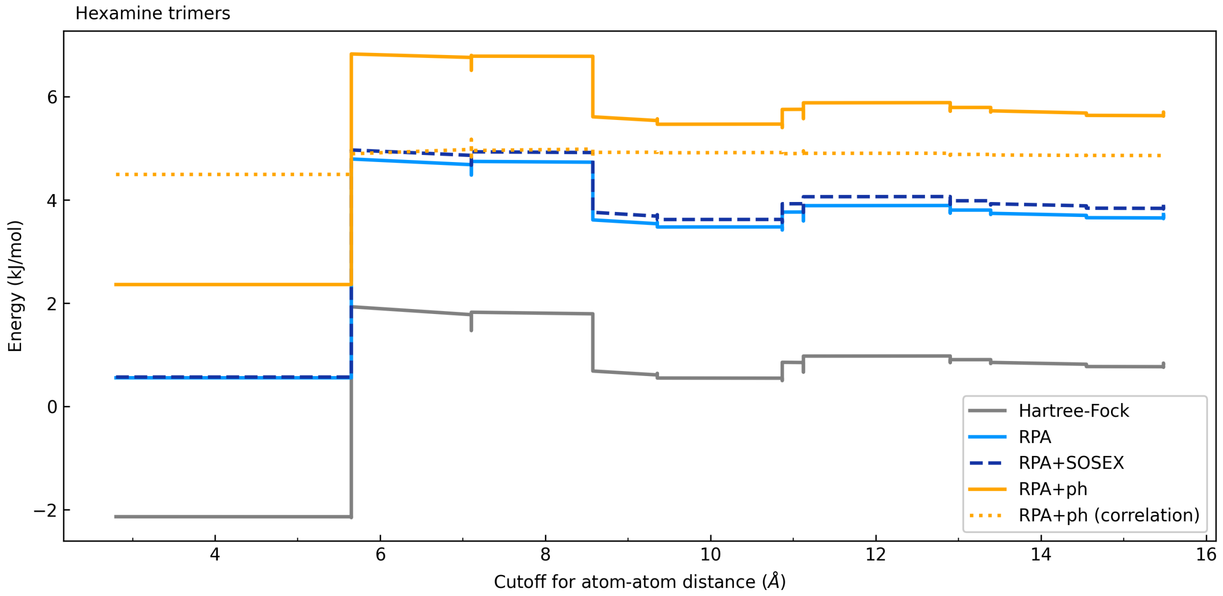

5.23 Succinic acid

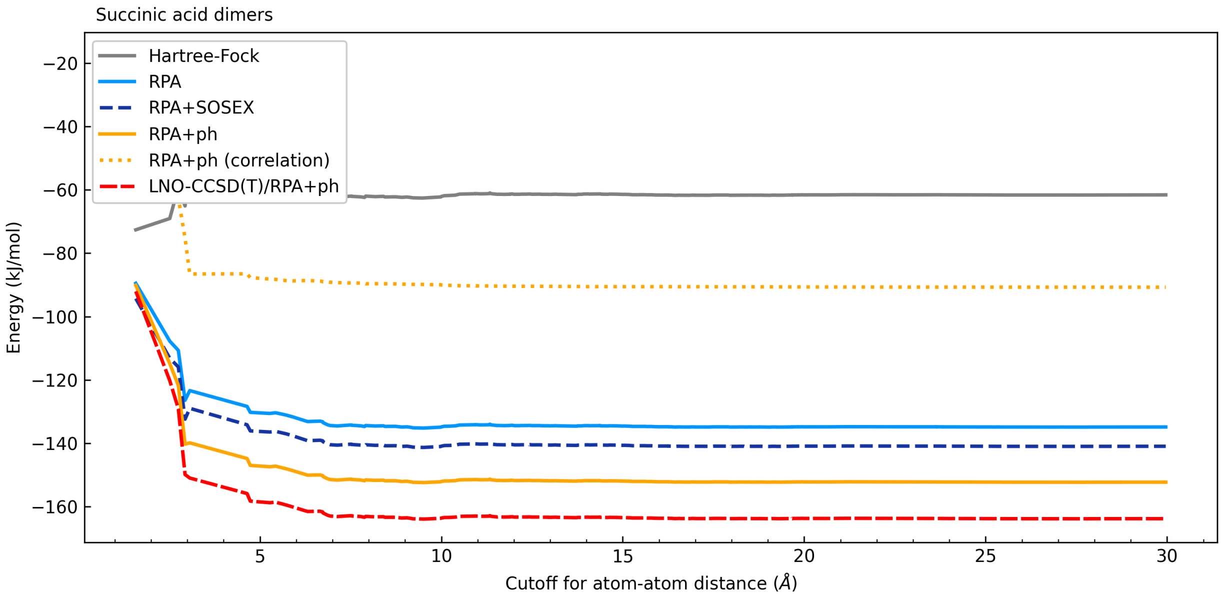

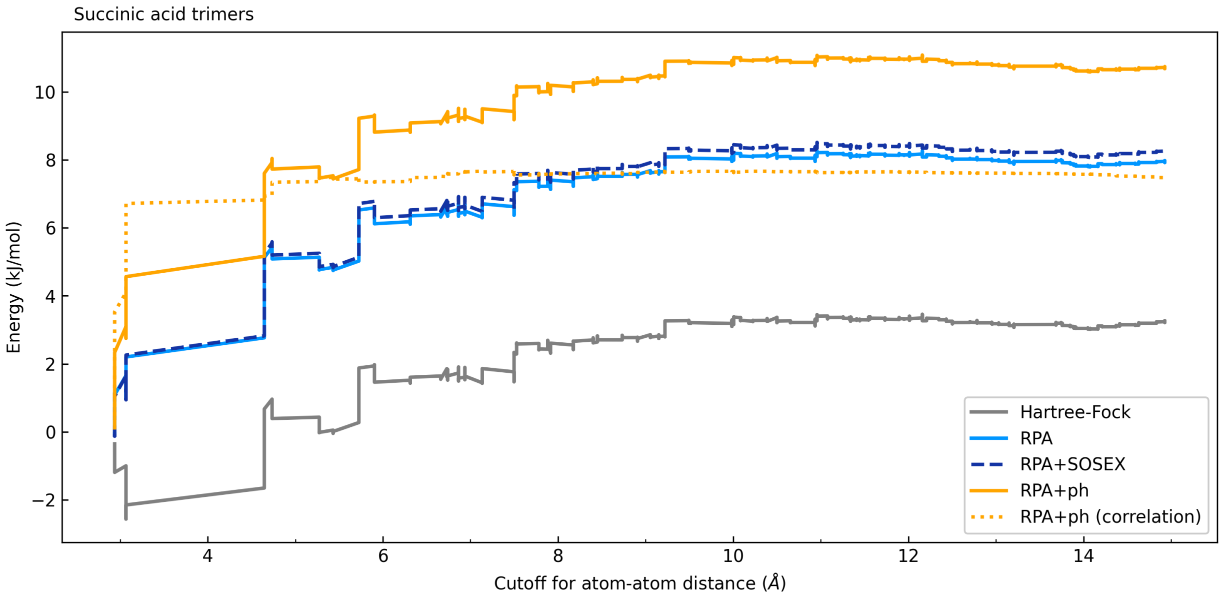

References

(S1) Deible, M. J.; Tuguldur, O.; Jordan, K. D. Theoretical Study of the Binding Energy of a Methane Molecule in a $(H_2O)_{20}$ Dodecahedral Cage. *J. Phys. Chem. B* **2014**, *118*, 8257–8263.

(S2) Modrzejewski, M.; Yourdkhani, S.; miga, S.; Klimes, J. Random-phase approximation in many-body

noncovalent systems: Methane in a dodecahedral water cage. *J. Chem. Theory Comput.* **2021**, *17*, 804–817.

(S3) Pham, K. N.; Modrzejewski, M.; Klimes, J. Assessment of random phase approximation and second-order Møller–Plesset perturbation theory for many-body interactions in solid ethane, ethylene, and acetylene. *J. Chem. Phys.* **2023**, *158*.

(S4) Sargent, C. T.; Metcalf, D. P.; Glick, Z. L.; Borca, C. H.; Sherrill, C. D. Benchmarking two-body contributions to crystal lattice energies and a range-dependent assessment of approximate methods. *J. Chem. Phys.* **2023**, *158*, 054112.

(S5) Borca, C. H.; Glick, Z. L.; Metcalf, D. P.; Burns, L. A.; Sherrill, C. D. Benchmark coupled-cluster lattice energy of crystalline benzene and assessment of multi-level approximations in the many-body expansion. *J. Chem. Phys.* **2023**, *158*, 234102.

(S6) Nelson, P. M.; Sherrill, C. D. Convergence of the many-body expansion with respect to distance cutoffs in crystals of polar molecules: Acetic acid, formamide, and imidazole. *J. Chem. Phys.* **2024**, *161*, 214105.

(S7) Della Pia, F.; Zen, A.; Alfè, D.; Michaelides, A. How accurate are simulations and experiments for the lattice energies of molecular crystals? *Phys. Rev. Lett.* **2024**, *133*, 046401.

(S8) Schuchardt, K. L.; Didier, B. T.; Elsethagen, T.; Sun, L.; Gurumoorthi, V.; Chase, J.; Li, J.; Windus, T. L. Basis Set Exchange: A Community Database for Computational Sciences. *J. Chem. Inf. Model.* **2007**, *47*, 1045–1052.

(S9) Halkier, A.; Klopper, W.; Helgaker, T.; Jorgensen, P.; Taylor, P. R. Basis set convergence of the interaction energy of hydrogen-bonded complexes. *J. Chem. Phys.* **1999**, *111*, 9157–9167.

(S10) Pham, K. N.; Modrzejewski, M.; Klimeš, J. Contributions beyond direct random-phase approximation in the binding energy of solid ethane, ethylene, and acetylene. *J. Chem. Phys.* **2024**, *160*.
